# Dynamic Association of NUP98 with the Human Genome

**DOI:** 10.1371/journal.pgen.1003308

**Published:** 2013-02-28

**Authors:** Yun Liang, Tobias M. Franks, Maria C. Marchetto, Fred H. Gage, Martin W. Hetzer

**Affiliations:** 1 Salk Institute for Biological Studies, Molecular and Cell Biology Laboratory, La Jolla, California, United States of America; 2 Salk Institute for Biological Studies, Laboratory of Genetics, La Jolla, California, United States of America; Northwestern University, United States of America

## Abstract

Faithful execution of developmental gene expression programs occurs at multiple levels and involves many different components such as transcription factors, histone-modification enzymes, and mRNA processing proteins. Recent evidence suggests that nucleoporins, well known components that control nucleo-cytoplasmic trafficking, have wide-ranging functions in developmental gene regulation that potentially extend beyond their role in nuclear transport. Whether the unexpected role of nuclear pore proteins in transcription regulation, which initially has been described in fungi and flies, also applies to human cells is unknown. Here we show at a genome-wide level that the nuclear pore protein NUP98 associates with developmentally regulated genes active during human embryonic stem cell differentiation. Overexpression of a dominant negative fragment of NUP98 levels decreases expression levels of NUP98-bound genes. In addition, we identify two modes of developmental gene regulation by NUP98 that are differentiated by the spatial localization of NUP98 target genes. Genes in the initial stage of developmental induction can associate with NUP98 that is embedded in the nuclear pores at the nuclear periphery. Alternatively, genes that are highly induced can interact with NUP98 in the nuclear interior, away from the nuclear pores. This work demonstrates for the first time that NUP98 dynamically associates with the human genome during differentiation, revealing a role of a nuclear pore protein in regulating developmental gene expression programs.

## Author Summary

Development of multicellular organisms such as humans requires appropriate activation of gene expression programs according to stages of differentiation. Many proteins that directly regulate this process have been identified, including histone-modifying enzymes and transcription factors. It is not clear whether nuclear pore proteins, proteins that form the only channels in the nuclear envelope that mediate nuclear transport, regulate developmental gene regulation in higher organisms such as humans. Here we show that one nuclear pore protein has a role in gene regulation during human cell differentiation, providing insight into the development-related and transport-independent function of nuclear pore proteins. We have found that the nuclear pore protein interacts with the human genome in a dynamic manner that is tightly linked to the developmental stage. In addition, manipulating the functional levels of the nuclear pore protein can disrupt expression of the developmental genes it associates with. Our results suggest that the nuclear pore protein functionally interacts with the genome during cell differentiation, uncovering an additional layer of developmental gene regulation in humans.

## Introduction

In eukaryotes, the nuclear envelope (NE) forms a membrane barrier around the nuclear genome. All molecular trafficking in and out of the nucleus is mediated by nuclear pore complexes, large multiprotein channels composed of ∼30 different nuclear pore proteins (Nups) that span the NE [1]–[3]. In addition to mediating transport, nuclear pore complexes have also been implicated in genome organization and transcriptional regulation [4]. Initial electron microscopy studies suggested that nuclear pore complexes specifically associate with decondensed, transcriptionally active euchromatin in an otherwise highly condensed, heterochromatic nuclear periphery [5]–[7]. Based on these observations, it has been proposed that nuclear pore complexes may interact with active genes to promote the export of their transcripts [7]. Consistent with this hypothesis, several reports have demonstrated that Nups bind active regions of the genome in *Saccharomyces cerevisiae* and more recently in *Drosophila melanogaster* [4], [8]–[17]. In yeast, all Nup-genome interactions identified so far are thought to occur at nuclear pore complexes at the nuclear periphery (i.e. ‘on-pore’ interaction). However, the organization of the nuclear pore complexes is highly dynamic [18] and a subset of mobile Nups has been shown to shuttle on and off nuclear pore complexes, thereby potentially extending the functional reach of Nups. Interestingly, evidence of intranuclear Nups that bind specific regions of the genome has been found in *Drosophila* suggesting that Nups can also bind chromatin away from the nuclear pores (i.e. ‘off-pore’ interaction) [8], [13], [17]. In *Drosophila* embryonic culture cells, Nups predominantly interacted with active genes inside the nucleoplasm, whereas the nuclear pore complexes at the nuclear periphery was associated with repressed genes [17].

Limited studies have been carried out to address whether Nups play an important role in transcription in the mammalian genome. In neonatal rat ventricular cardiomyocytes, NUP155 was found to interact with the histone deacetylase HDAC4 and nuclear pore components associate with a number of HDAC4-target genes [19]. The only study that addressed the potential role of Nups in gene regulation in human cells has shown that nuclear pore complexes preferentially associate with repressive chromatin domains [20]. Combined with studies from fungi and flies, it appears that Nups can interact with both active and silent loci, depending on the cell type or the type of Nups investigated. Therefore, it is tempting to speculate that Nups may dynamically associate with the genome according to developmental stages during differentiation. Accumulating evidence suggests that the organization of the genome is highly dynamic during development [21]–[23]. For example, on a global level, the hyperdynamic and open chromatin organization has been correlated to the differentiation potential of pluripotent cells, and this property is lost upon differentiation. Moreover, on the single-gene level, repositioning of developmental genes and tissue-specific promoters relative to the nuclear periphery during differentiation has been well documented [24]–[30]. The potential involvement of Nups in chromatin-related aspects of developmental regulation is further supported by the findings that mutations in multiple Nups caused specific developmental defects rather than a global deficiency that would have been predicted if the sole role of Nups was to mediate transport in all cell types [31].

Several studies suggest that Nups are involved in developmental gene regulation in lower organisms. In yeast, the mating pheromone alpha factor induces alterations in the association between Nups and specific genomic regions [9]. In *Drosophila* salivary glands, a subset of Nups including the mobile NUP98 can dissociate from nuclear pores and activate a number of ecdysone-induced genes in the nuclear interior (i.e. ‘off-pore’ Nup-gene interaction). These findings raise several key questions regarding the chromatin-related function of Nups during development. For instance, are Nups involved in establishing gene expression programs in diploid cells of mammalian organisms, especially human, during differentiation of pluripotent cells and establishment of cell fate? Do Nups relocate to developmentally induced genes on a genome-wide level in human cells? What are the differences between ‘on-pore’ and ‘off-pore’ Nup-gene interactions in the context of development, and do nuclear pores at the nuclear periphery have a role in developmental gene regulation?

We decided to determine if NUP98, a nuclear pore complex component that is located on both the cytoplasmic and the nucleoplasmic faces of the nuclear pore complex and has the capacity to move on and off the nuclear pore [18], [32], interacts with the human genome. Using chromatin immunoprecipiation sequencing (ChIP-seq) we show that NUP98 associates with developmentally regulated genes in stem cells and progenitor cells. In neural progenitor cells, overexpression of full-length NUP98 increases expression levels of a subset of its binding targets, and overexpression of a dominant negative fragment of NUP98 decreases mRNA levels of NUP98-associated genes. In addition, we found that developmental NUP98-gene interactions occur both on nuclear pore complexes and in the nuclear interior. The ‘on-pore’ interactions seem to be enriched for genes involved in the initial stage of developmental induction, whereas the ‘off-pore’ (i.e. intranuclear) targets are comprised of genes mediating later stages of developmental induction. We concluded that during human stem cell differentiation, NUP98 associated with specific regions of the genome in a manner that was tightly coupled to the developmental stage. In addition, the nuclear pores appeared to function as a transient platform that supported the initial induction of developmental genes.

## Results

### NUP98 binds to distinct genomic regions in different human cell types

**Figure 1:**
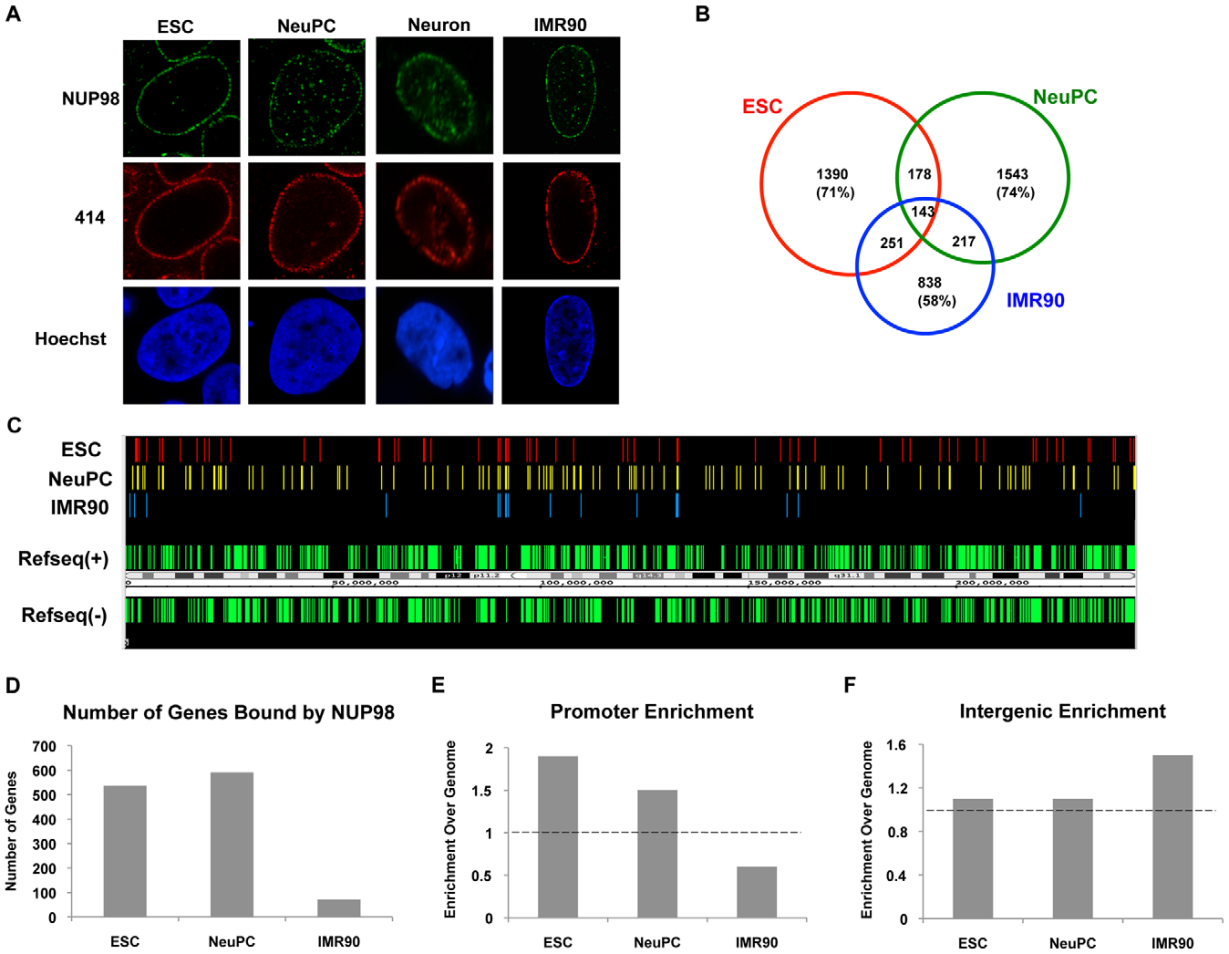
NUP98 binds to distinct genomic regions in cells of different developmental stages. (A) Human embryonic stem cells (ESC), neural progenitor cells (NeuPC), neurons, and IMR90 cells were stained with anti-human NUP98 antibodies (green), mAb414 (red), and Hoechst (blue). (B) Venn diagram of the overlap between NUP98-binding regions in human embryonic stem cells (ESC), neural progenitor cells (NeuPC), and lung fibroblast cells (IMR90). (C) Chromosomal view of NUP98 binding regions on chromosome 1 in ESCs, NeuPCs, and IMR90 cells. Refseq(+) indicates Refseq genes on the (+) strand, and Refseq(−) indicates Refseq genes on the (−) strand. (D) Number of genes bound by NUP98 in ESCs, NeuPCs, and IMR90 cells. (E) Promoter enrichment of NUP98 binding regions in ESCs, NeuPCs, and IMR90 cells. The percentage of NUP98 binding regions that overlap with promoters was normalized against the percentage of promoters in human genome. (F) Intergenic enrichment of NUP98 binding regions in ESCs, NeuPCs, and IMR90 cells. The percentage of NUP98 binding regions that overlap with intergenic regions was normalized against the percentage of intergenic regions in human genome.

To test whether NUP98 can bind to the mammalian genome during cell differentiation, we performed ChIP-Seq experiments on cultured human embryonic stem cells (ESCs), neural progenitor cells (NeuPCs) that were differentiated in vitro from ESCs, and neurons that were differentiated in vitro from NeuPCs. We also determined the presence of chromatin-bound NUP98 in lung fibroblast IMR90 cells as an example of another differentiated cell type. As expected, in all four cell types, NUP98 was found both on nuclear pores at the nuclear periphery and intranuclear sites, consistent with its reported capacity to move on and off the nuclear pores (Figure 1A) [18], [32]. We first validated the ChIP-Seq method using IMR90 cells with two independent antibodies against human NUP98. As expected, both antibodies stained nuclear pores and a few intranuclear sites in IMR90 cells (Figure S1A). Additionally, both proved efficient and specific in western blot and immunoprecipitation experiments (Figure S1B, S1C). Since Nups were not expected to bind directly to DNA, we employed two cross-linking conditions for the ChIP-Seq experiment, formaldehyde single cross-linking and formaldehyde-disuccinimidyl glutarate double-crosslinking in order to more efficiently crosslink indirect Nup-chromatin contacts. After crosslinking, we immunoprecipitated NUP98 using the two antibodies, purified DNA that was immunoprecipitated, and had DNA amplified and subjected to deep sequencing. Sequencing reads were then mapped to the human genome (Figure S2). The results from the four ChIP-Seq experiments, using two antibodies and two cross-linking conditions, were highly consistent (Figure S1D–S1F), with 73% NUP98-binding regions from pull-down using the first antibody overlapping with 78% NUP98-binding regions using the second antibody. We further validated our results by randomly selecting several NUP98-binding regions called from the ChIP-Seq experiment and confirming the interaction between NUP98 and these regions by ChIP-qPCR (Figure S1G).

After validation of the ChIP-Seq method, we extended the ChIP-Seq analysis to human embryonic stem cells, human embryonic stem cell-derived NeuPCs that were ∼90% positive for the neural progenitor cell marker Nestin (Figure S3), and postmitotic neurons. Interestingly, the genome-binding pattern of NUP98 varied greatly depending on the developmental stage of the cells. NUP98 occupied more genomic regions in ESCs and NeuPCs than in differentiated cells. Further analysis revealed that 71% of NUP98-chromatin sites in ESCs and 74% in NeuPCs were specific for the respective cell-type (i.e. not found in the other cell types) (Figure 1B, 1C). The most dramatic difference was found in neurons where essentially no significant enrichment for NUP98 binding could be identified (Figure S4 and data not shown). Together, these findings suggest that Nup98's ability to interact with the human genome is developmentally regulated.

We further analyzed whether NUP98-DNA interaction occurred on gene regulatory elements and/or coding regions in ESCs and NeuPCs by assigning NUP98 binding regions to promoters, exons, introns, and intergenic regions. In both ESCs and NeuPCs, NUP98 bound to 500–600 genes (Figure 1D) and exhibited a significant enrichment in promoter regions (Figure 1E). It is important to note that the few NUP98 binding sites in IMR90 cells were preferentially found in intergenic regions (Figure 1F), providing additional evidence for a dynamic and developmentally-controlled association of NUP98 with the human genome. Although we cannot rule out that NUP98 binding in IMR90 has functional significance, we decided to focus our analysis on NUP98-bound genes in ESCs and NeuPCs.

### NUP98 associates with conserved DNA motifs

**Figure 2:**
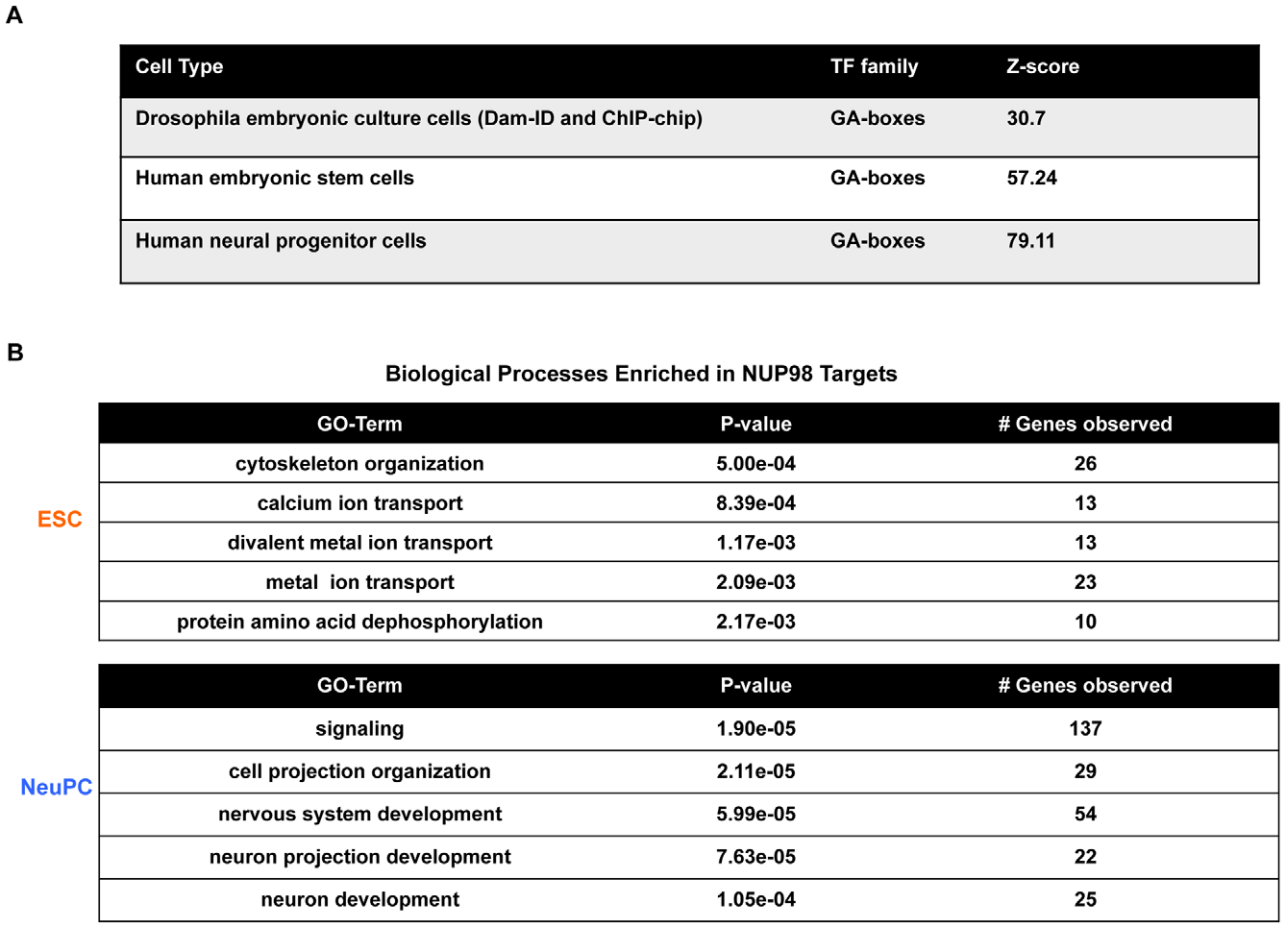
Transcription factor motif and gene ontology analysis of NUP98 binding regions. (A) GA-boxes is an over-represented transcription factor motif in *Drosophila* from published NUP98 Dam-ID and ChIP-chip datasets [8], [17] and in human ESCs and NeuPCs. Z-score represents the distance from the population mean in units of the population standard deviation. (B) Biological processes enriched in NUP98 binding genes in ESCs and NeuPCs by gene ontology analysis.

In order to identify potential DNA sequence motifs and/or potential NUP98-interacting transcription factors that direct NUP98-DNA binding, we analyzed the transcription factor motifs overrepresented in NUP98-binding sequences found in ESCs and NeuPCs. We found that GA-boxes were an evolutionarily conserved NUP98-associated motif. This motif was not only overrepresented in human NUP98-binding genomic regions, but also in published *Drosophila* NUP98 binding sequences (Figure 2A, Figure S5A and S5B) [8], [17]. In *Drosophila*, GA-boxes are recognized by GAGA factor, which is a transcriptional activator that is crucial for the proper expression of several homeotic genes [33]. This suggests that the interaction between NUP98 and GA-box motifs, potentially related to the regulation of developmental genes, is evolutionary conserved and further validates our ChIP-Seq results.

We also identified YY1 binding site as NUP98-associated motif in ESCs and NeuPCs (Figure S5C). Both GAGA factor and YY1 have been linked to boundary activities, in line with the potential role of Nups in the compartmentalization of chromatin into active and silent domains [31], [34]–[36]. The binding motif of nuclear DEAF-1 related (NURD)/homolog to *Drosophila* DEAF-1 is also a NUP98-associated motif enriched in both ESCs and NeuPCs (Figure S5C). NURD displays homology to the protein SP100, a component of the promyelocytic leukemia-associated nuclear body, implying that NUP98 might be involved in the regulation of nuclear bodies and is consistent with the reported link of NUP98 to leukemia [37]–[39].

Moreover, we have found that in ESCs specifically, NUP98 binding sequences were enriched for motifs recognized by GC-Box factors SP1, C2H2 zinc finger transcription factors and SMAD (Figure S5C). These findings raise the exciting possibility that NUP98 is linked to the core transcription circuitry that is crucial for the maintenance of pluripotency in ESCs [40], [41].

### NUP98 interacts with neural developmental genes specifically in neural progenitor cells

To further understand the dynamic DNA-binding behavior of NUP98, we investigated the functional categories of genes bound by NUP98 in ESCs and NeuPCs by gene ontology analysis. In ESCs, the top functional category enriched in NUP98 targets was found to be cytoskeleton organization (Figure 2B). This is consistent with recent reports showing that in Drosophila embryonic culture cells NUP98 binding targets were also enriched for cytoskeleton genes [17]. As discussed later (Figure S7), NUP98 targets in ESCs could be divided into two groups, one associated with active histone marks and one associated with silent histone marks. The active group of NUP98 targets in human ESCs was enriched for genes in the functional categories of cell cycle regulation, cell communication and metabolism. Such genes were also enriched in Drosophila NUP98 targets in embryonic cells [17] (and data not shown).

Interestingly, NUP98 targets were specifically enriched for neurogenesis genes in NeuPCs, including genes in functional categories of nervous system development, neuron projection development, and neuron development (Figure 2B). Examples of NUP98-interacting neurogenesis genes include NRG1, ERBB4, SOX5, and ROBO [42]–[44]. Furthermore, analysis of disease terms enriched in NUP98 targets in NeuPCs revealed that NUP98 is linked to genes involved in multiple diseases of the nervous system (Figure S6). Such diseases include neurodegenerative disorders such as Alzheimer disease and tumors such as glioma and neoplasms of the nerve tissue. The latter finding might be relevant for the previously reported role of NUP98 in tumorigenesis [39]. These results suggest that NUP98 is recruited to neural developmental genes in a developmentally controlled manner.

### NUP98 binding correlates with developmental gene expression in neural progenitor cells

**Figure 3:**
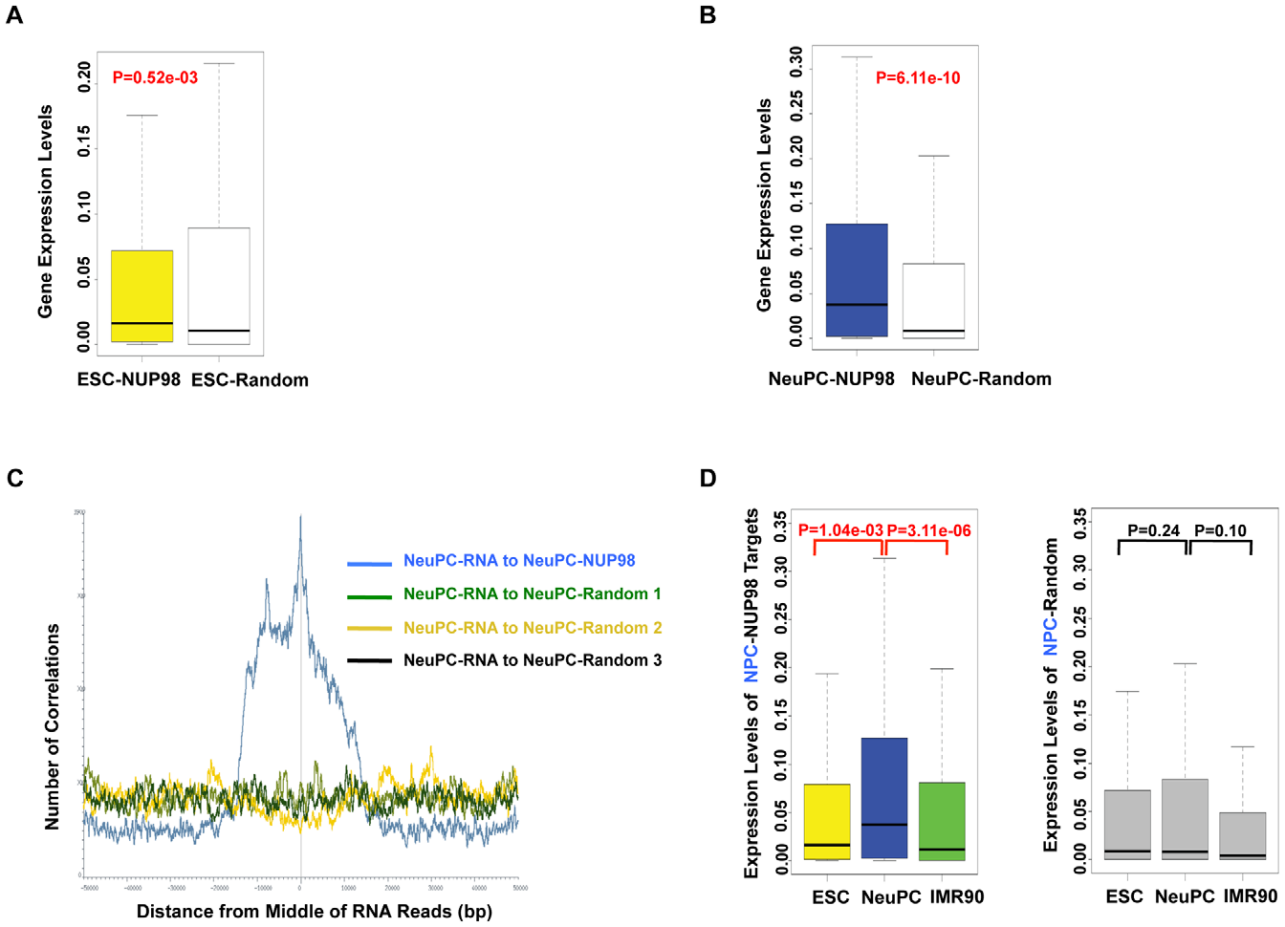
NUP98 binding correlates with developmental gene expression in neural progenitor cells. (A, B) Expression levels of NUP98 binding genes (-NUP98) and same number of randomly selected genes (-Random) in embryonic stem cells (ESC-) (A) and neural progenitor cells (NeuPC-) (B) were plotted. P value was obtained by Mann-Whitney U tests. Randomization was conducted for at least 10 times and similar results were obtained (data not shown). Gene expression values were obtained from [42], [43]. Top and bottom of the boxes in the plot are 25^th^ and 75^th^ percentile, centerline is the 50^th^, and whiskers extend to 1.5 interquartile range from the upper and lower quantile. (C) Positional correlation between expressed mRNA and NUP98-binding (blue) or three sets of same number- and size-matched, randomly selected regions (green, yellow, and black) in NeuPCs. mRNA expression data were from [43]. (D) Expression level change of neural progenitor cell-NUP98 targets during development, i.e. in ESCs, NeuPCs, and IMR90 cells (left). All NUP98 binding regions detected in neural progenitor cells that overlap with genes (promoters/exons/introns) were used. Expression level change of same number of randomly selected genes during development, i.e. in ESCs, NeuPCs, and IMR90 cells, were shown as negative control (right). Randomization was conducted for at least 10 times and similar results were obtained (data not shown). P values were obtained by Mann-Whitney U tests. Gene expression values were obtained from [42], [43]. Top and bottom of the boxes in the plot are 25^th^ and 75^th^ percentile, centerline is the 50^th^, and whiskers extend to 1.5 interquartile range from the upper and lower quantile.

The specific association between NUP98 and neurogenesis genes in NeuPCs raised the possibility of a positive correlation between NUP98 binding and the activation of these genes during neural differentiation. To test this possibility, we compared the expression levels of genes bound by NUP98 to those of the same number of randomly selected genes in ESCs and NeuPCs using published RNA-Seq datasets [42], [43] (Figure 3A, 3B). We found that genes bound by NUP98 had higher expression levels in NeuPCs compared to randomly selected gene sets, suggesting that NUP98-binding was associated with elevated gene expression levels. As an independent test, we correlated the genomic localization of NUP98-binding regions to that of expressed mRNA in NeuPCs (Figure 3C). We were able to detect a positive correlation between the location of NUP98 binding on the genome and the location of mRNA production, indicating the positive correlation between NUP98 binding and mRNA expression.

Having established a link between NUP98 binding and active gene expression in NeuPCs, we asked if NUP98 binding to its target genes in NeuPCs would coincide with their transcriptional induction during neural differentiation. We found that NUP98-bound loci in NeuPCs had higher expression levels than either ESCs or IMR90 cells (Figure 3D). By contrast, for randomly selected genes, there was no statistically significant difference in expression levels in any of the analyzed cell types. Together, these findings support the notion that NUP98 gains association with developmental genes as they are undergoing transcriptional activation during development.

Considering all genes in the human genome, from published RNA-Seq datasets, there are a total of 8388 genes activated during differentiation of ESCs into NeuPCs. They were defined as genes whose expression levels were not detectable in ESCs but detectable in NeuPCs or were upregulated by more than two-folds in NeuPCs compared to ESCs [42], [43]. 2.7% of these genes gained NUP98 binding in NeuPCs compared to ESCs, suggesting that NUP98 is associated with specific subset of developmentally regulated genes.

In addition, we found 138 genes that lost NUP98 binding and also became inactivated in terms of expression levels upon differentiation from ESCs to NeuPCs. The expression levels of these genes were detectable in ESCs but undetectable in NeuPCs or were downregulated more than two-fold in NeuPCs compared to ESCs from published RNA-Seq datasets [42], [43]. This suggests that NUP98 might also be linked to active gene expresison in pluripotent cells.

### NUP98 loses association with active chromatin domains in post-differentiation IMR90 cells

**Figure 4:**
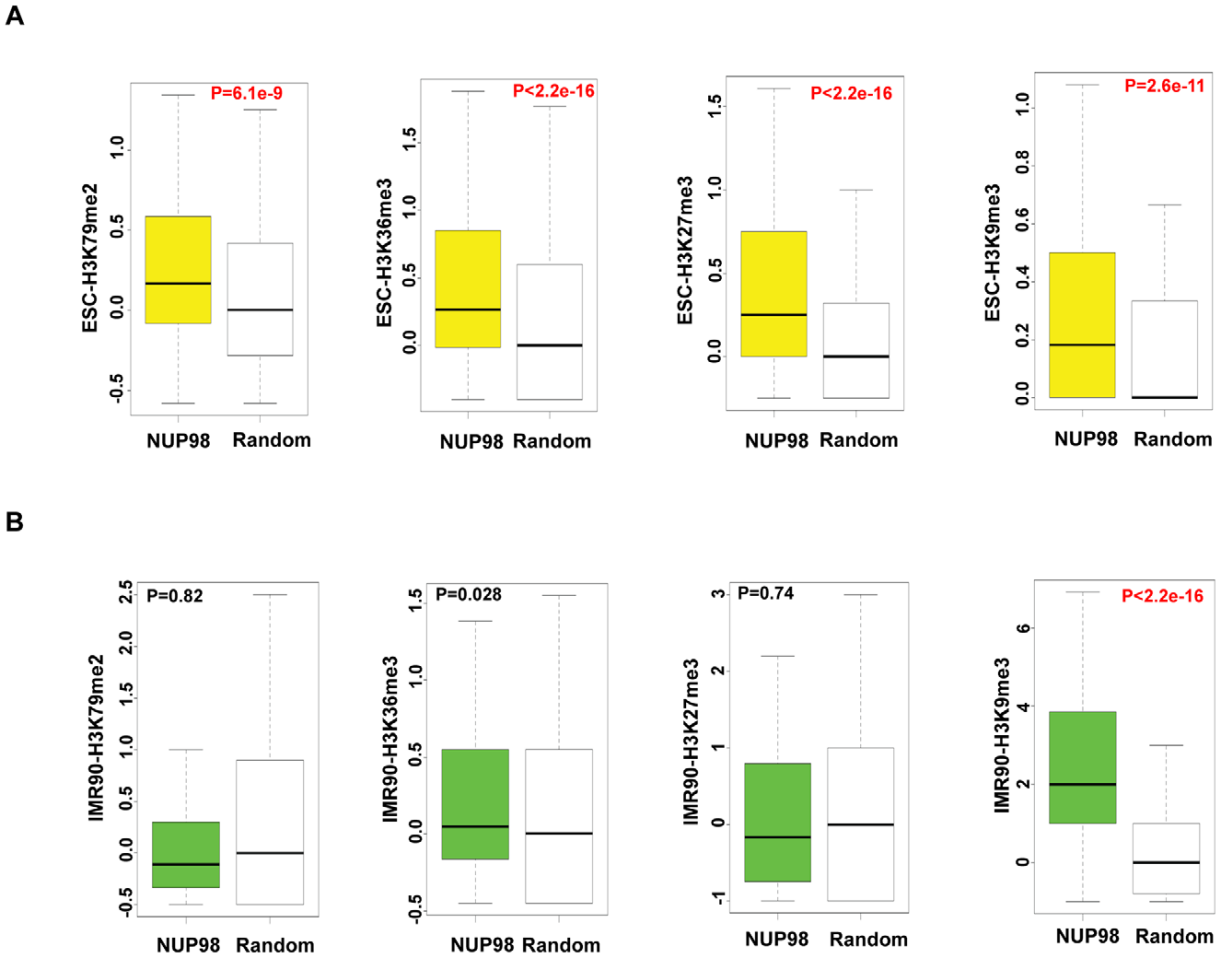
NUP98 loses association with active chromatin domains in post-differentiation IMR90 cells. Histone modification levels of NUP98 binding genes (-NUP98) and same number of randomly selected genes (-Random) in embryonic stem cells (ESC-) (A) and lung fibroblasts (IMR90-) (B) were plotted. P values were obtained by Mann-Whitney U tests. Randomization was conducted for at least 10 times and similar results were obtained (data not shown). Histone modification levels were calculated from [42], GSM605321, and GSM605309. Top and bottom of the boxes in the plot are 25^th^ and 75^th^ percentile, centerline is the 50^th^, and whiskers extend to 1.5 interquartile range from the upper and lower quantile.

In contrast to the direct correlation between NUP98 binding and gene activation in NeuPCs, the scenario in ESCs appears more complicated. To gain additional insight into the type of chromatin environment that NUP98 interacts with, we compared NUP98 binding to the levels of different histone modifications by comparing our ChIP-Seq datasets to published ChIP-Seq datasets of histone modifications in ESCs [42]. Specifically, we examined H3K79me2 and H3K36me3 that are linked to active transcription, as well as H3K27me3 and H3K9me3 that are linked to repressed chromatin domains [44]. We compared histone modification levels for NUP98-binding regions and randomly selected regions as negative controls. We found that, in ESCs, NUP98 binding showed positive correlation with both active and silent histone marks. In contrast, NUP98 binding in IMR90 cells, which does not target promoter regions, was exclusively linked to high H3K9me3 levels (Figure 4). This observation is consistent with the idea that NUP98 is preferentially, if not exclusively, involved in developmental gene regulation in pluri-/multi-potent cells whereas in differentiated cells either associates with repressive chromatin (e.g. IMR90 cells) or lacks chromatin association altogether (e.g. neurons).

The finding that NUP98 associates with both active and silent chromatin domains in ESCs could be due to two reasons: 1) NUP98 is directed to bivalent domains that exhibit both active and silent histone marks or 2) there are two subsets of NUP98 targets, one active and one silent. To distinguish between these two possibilities, we determined the extent to which pairs of histone marks were found at NUP98 binding regions by Pearson's Correlation analysis (Figure S7A). Specifically, we examined the extent of correlation between 4 pairs of histone marks, H3K36me3 (active histone mark) - H3K27me3 (silent histone mark), H3K36me3 (active) -H3K9me3 (silent), H3K79me2 (active) - H3K27me3 (silent), and H3K79me2 (active) - H3K9me3 (silent). The result showed that the correlation between active and silent histone marks for NUP98 targets was low, suggesting NUP98-binding regions can be divided into at least two distinct subgroups, the group with active histone marks and the group with silent marks. In order to examine the types of genes included in each group, for each histone mark we ranked the genes bound by NUP98 by the levels of the histone mark found at that loci, selected the top 40% of the genes and performed gene ontology analysis on those genes (Figure S7B–S7D). We found that NUP98 targets with high levels of active histone marks (H3K79me2 or H3K36me3) were uniquely enriched for genes involved in macromolecule and nucleic acid metabolism and various aspects of the cell cycle such as nuclear division and mitosis. On the other hand, NUP98 targets, which were characterized by high levels of repressive histone mark H3K27me3, were uniquely enriched for genes involved in transmembrane transport. Furthermore, we confirmed that NUP98 targets with high levels of active histone marks were actively transcribed, whereas the ones with high levels of silent histone marks were repressed (Figure S7E–S7H). These observations are reminiscent of the findings in Drosophila embryonic culture cells in which NUP98 associates with both active and repressed genes as well as cell cycle and nucleic acid metabolism genes ([17]; (data not shown). Combining the observations in Drosophila and human cells, it is possible that NUP98 exhibits an evolutionally conserved role in associating with and potentially regulating cell cycle and nucleic acid metabolism genes.

Together these data suggest that in undifferentiated ESCs, NUP98 associates with one subgroup of active genes including cell cycle and nucleic acid metabolism genes as well as with one group of silent chromatin regions.

### NUP98 functionally associates with genes involved in neural development

**Figure 5:**
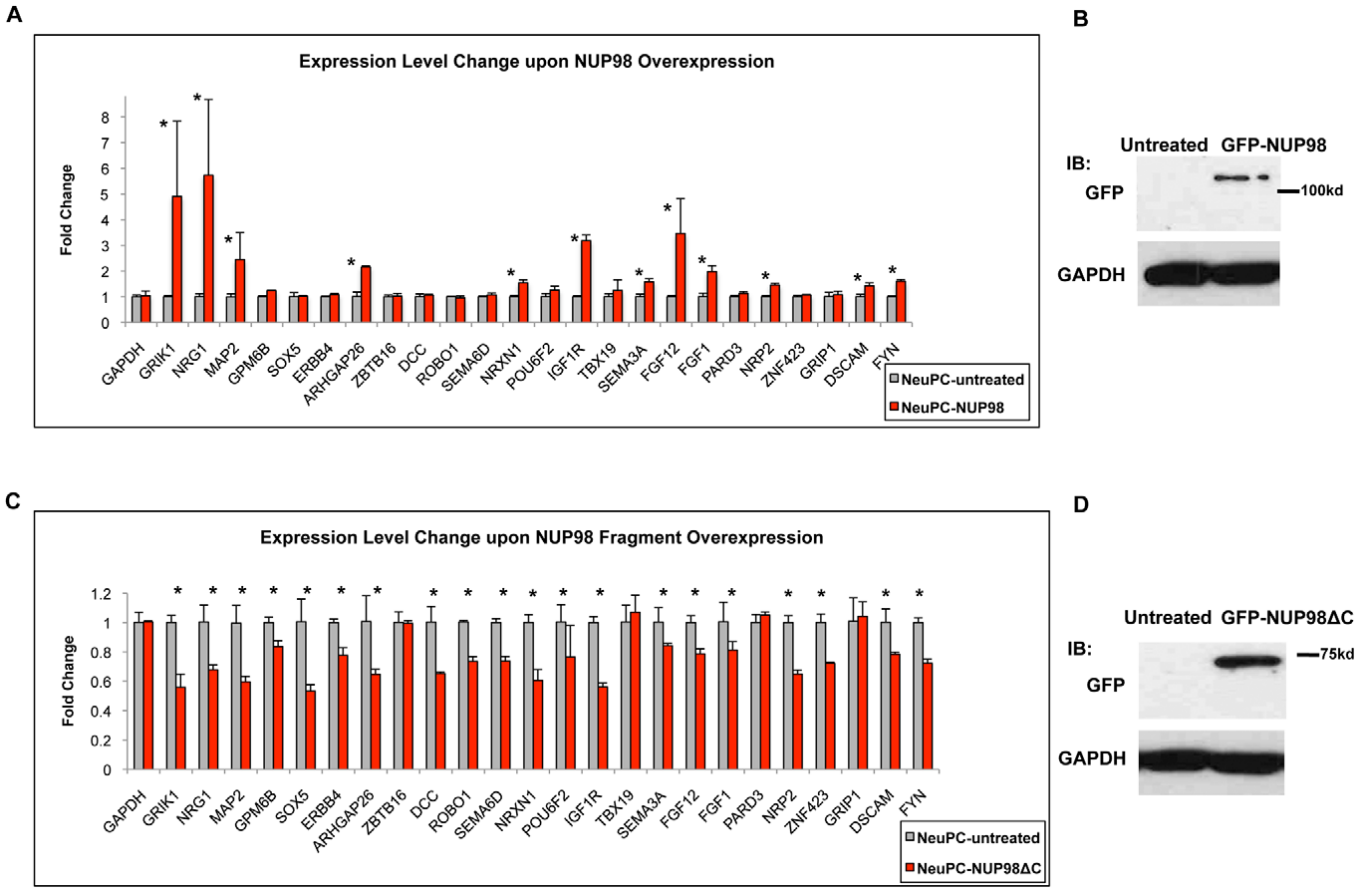
NUP98 is functionally relevant for the expression of its binding targets. (A) Fold change in expression levels upon full length NUP98 (-NUP98) overexpression in NeuPCs. Error bars were computed as standard deviation from triplicates. P value was obtained from Student's t-test and comparisons with P value<0.05 indicated with asterisks. (B) Western blot GAPDH and GFP in NeuPCs with overexpression of GFP-NUP98 or untreated condition as negative control. (C) Fold change in expression levels upon NUP98 fragment (-NUP98ΔC) overexpression in NeuPCs. Error bars were computed as standard deviation from triplicates. P value was obtained from Student's t-test and comparisons with P value<0.05 indicated with asterisks. (D) Western blot of GAPDH and GFP in NeuPCs with overexpression of GFP-NUP98 fragment (GFP-NUP98ΔC) or untreated condition as negative control.

Since NUP98 associated with neural development genes during neural differentiation, we asked if this nuclear pore complex component plays a role in their expression. We randomly selected 24 genes from the 54 genes in the ‘nervous system development’ gene ontology category that showed specific enrichment in NeuPCs (Figure 2B) together with GAPDH as well as additional genes that did not bind NUP98 as negative controls, and examined how their expression levels were affected by NUP98 overexpression in neural progenitor cells using qRT-PCR (Figure 5A, 5B, Figure S8A). To do this, NeuPCs were transfected with NUP98 and the overexpressed NUP98 localized to both nuclear pores and nucleoplasm (Figure S9). Strikingly, we found that 12 NUP98-associated neural developmental genes showed statistically significant increase in expression levels upon NUP98 overexpression, indicating that NUP98 regulates the transcription of these genes. Since not all genes responded to NUP98 overexpression, we suspect that NUP98 might not be rate-limiting in all its target genes. We then overexpressed a fragment of NUP98 (amino acid 1–504) in NeuPCs, which lacks a C-terminal domain of NUP98 that is no longer capable of binding to the nuclear pore complex (Figure S9). We were interested in this region of NUP98 because this is the same fragment as appeared in multiple NUP98-involved leukemic fusions and this fragment has been found to interfere with the differentiation of haematopoietic progenitor cells [39]. Given reported evidences for a role of NUP98 in gene regulation [8], [17] and our observation of the association between NUP98 and developmental genes at the progenitor cell stage, we hypothesized that this NUP98 fragment might interfere with the expression of NUP98 targets required for neural differentiation. We found that overexpression of this fragment of NUP98 had a dominant negative effect on the expression of NUP98-binding neural developmental genes, and 20 of the 24 genes exhibited statistically significant decrease in expression levels (Figure 5C, 5D). No significant effects on gene expression have been observed for GAPDH as well as additional genes that did not bind NUP98 (Figure 5C, 5D, Figure S8B). This suggests that the C-terminal domain of NUP98 is required for the expression of NUP98 target genes because the fragment lacking this domain could not stimulate expression of target genes as the full length NUP98 protein did. As an additional negative control, we overexpressed NUP35 using the same vector and found no effects on the expression of NUP98-binding genes (Figure S10). We did not examine the effect of NUP98 knockdown on gene expression because NUP98 is encoded on the same mRNA with a core component of the nuclear pore, NUP96, which is essential to nuclear pore biogenesis [32]. Knockdown of NUP98 causes simultaneous knockdown of NUP96 and a failure in nuclear pore formation and cell death (data not shown). Therefore, it was not possible to specifically analyze the gene regulatory function of NUP98 from such knockdown experiments.

Collectively, these results indicate that NUP98 is functionally relevant for the expression of neural developmental genes it associates with in NeuPCs.

### Expression level changes of NUP98 targets during neural differentiation

**Figure 6:**
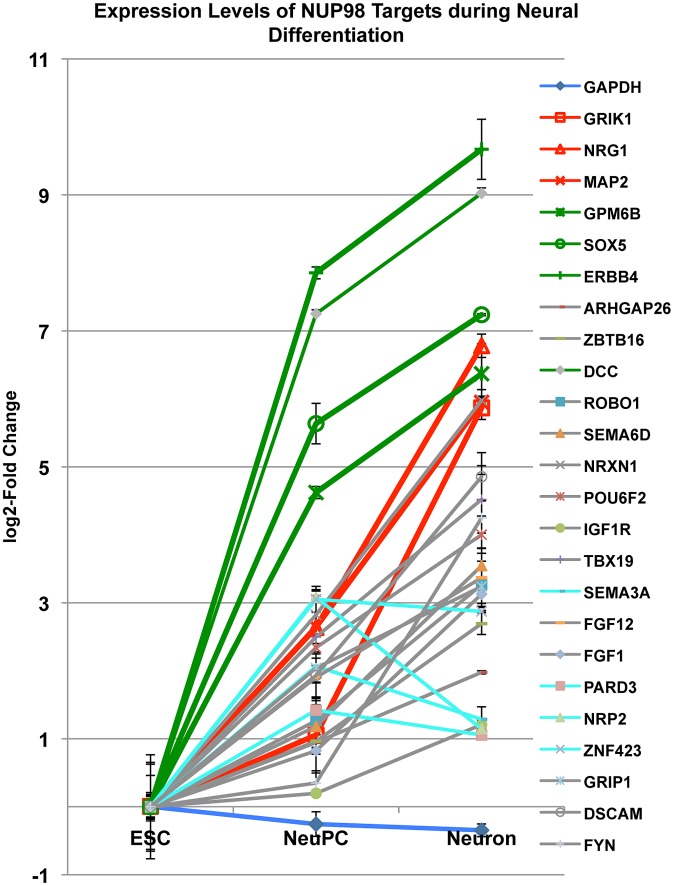
Expression level changes of NUP98 target genes during neural differentiation. Fold change in expression levels of neural progenitor cell-NUP98 binding genes from embryonic stem cells (ESC) to neural progenitor cells (NeuPC) and to neurons (Neuron) on a log_2_-scale. Different groups of developmentally regulated NUP98 binding genes were labeled in red, green, aqua or grey. Error bars were computed as standard deviation from triplicates.

To obtain further insights into the role of NUP98 during differentiation we monitored the mRNA levels of 24 NUP98 target genes that were in the neural development gene ontology category through differentiation from ESCs to NeuPCs, and subsequently to postmitotic neurons in which Nup98 does not seem to bind the genome (Figure 6). We found that all 24 genes were upregulated when ESCs were differentiated to NeuPCs, consistent with the genome-wide correlation analysis and supporting a role of NUP98 in the induction of transcription (Figure 3D). When NeuPCs were further differentiated to neurons, the majority of genes (20 genes) showed continued transcriptional induction. Among those 20 genes, we focused on 6 genes that exhibited the most dramatic increase in expression in neurons. We observed that these genes could be largely divided into two groups (Figure 6). Group I genes (GRIK1, NRG1, and MAP2; colored in red) showed modest transcriptional induction in NeuPCs compared to ESCs. However, this cohort of genes underwent a robust increase in expression during the transition from NeuPCs to neurons. Group II genes (GPM6B, SOX5, and ERBB4; colored in green) underwent a dramatic activation in the initial commitment of ESCs to NeuPCs and only slight upregulation during subsequent neuronal differentiation. This suggests that NUP98 associates with both genes starting to be developmentally induced (Group I genes) and genes that are at a later stage of induction (Group II genes) in NeuPCs.

### Group I and group II NUP98 targets exhibit distinct localization of genes

**Figure 7:**
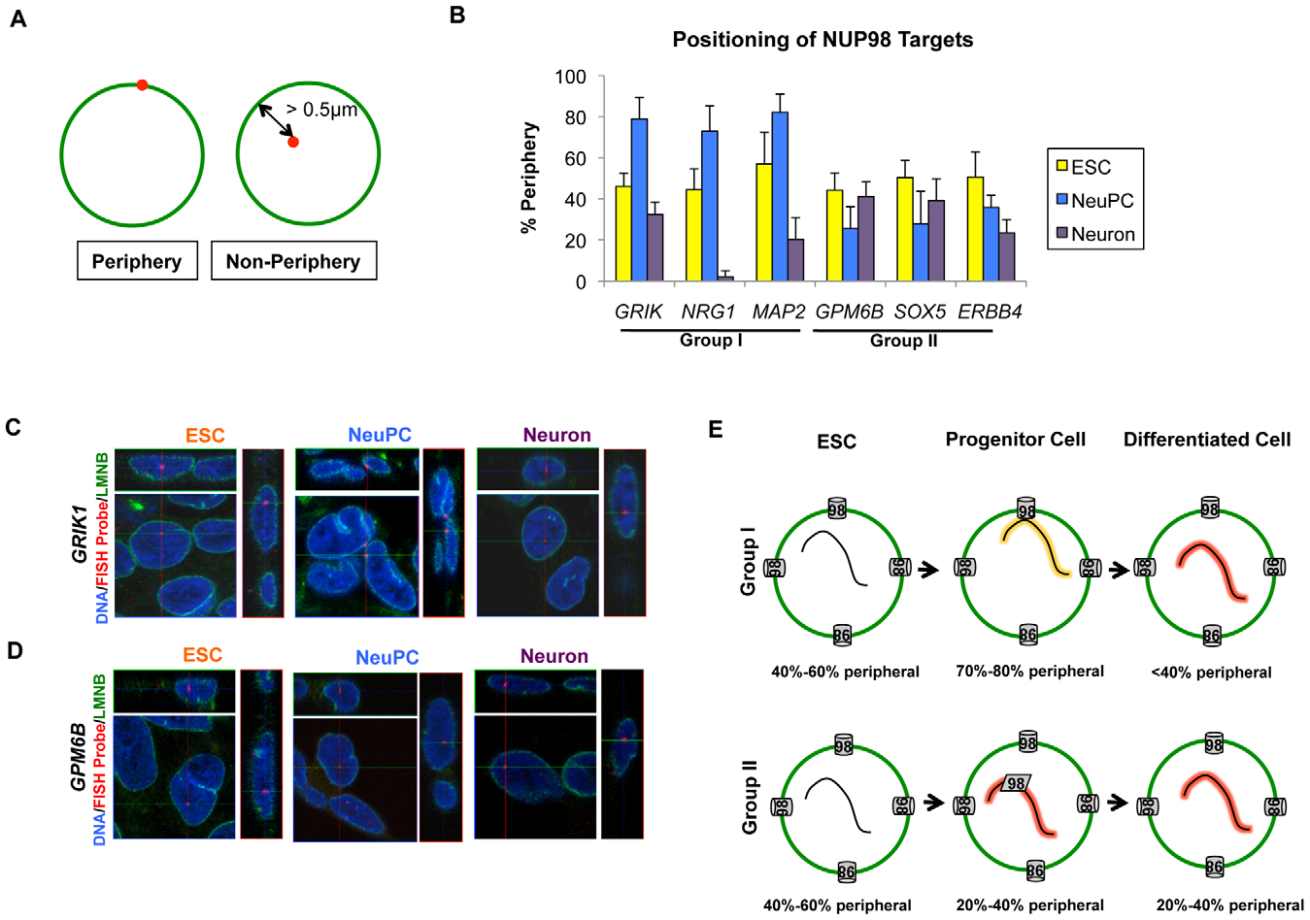
Distinct localization of two groups of NUP98-regulated developmental genes. (A) Criteria for counting gene localization as ‘Periphery’ or ‘Non-Periphery’, with LMNB staining in green and FISH signal in red. Genes counted as ‘Periphery’ were localized within 0.5 µm of the nuclear lamina. (B) Percentage of periphery localization of NUP98 binding genes through development, i.e. in ESC (yellow), NeuPC (blue) and Neuron (purple), determined from IF-FISH experiments. Error bars were calculated as standard deviation from triplicates for a total of at least 100 cells using 3D reconstruction of images. (C, D) Representative 3D IF-FISH images showing the localization of (C) group I genes (*GRIK1*) and (D) group II genes (*GPM6B*) through development, i.e. in ESC, NeuPC, and Neuron. FISH probes were shown in red, LMNB staining in green, and Hoechst in blue. Each set of images includes the x-y, y-z and x-z planes that cross at the FISH probe signal. (E) Model of two groups of NUP98-gene interaction. Group I genes are at the beginning stage of developmental induction in neural progenitor cells and interact with NUP98 at the nuclear pores in the NE, and subsequently translocate to intranuclear sites upon full induction in neurons. In contrast, group II genes are already greatly activated in NeuPCs and interact with NUP98 at intranuclear sites away from the NE. The percentage of genes observed at the nuclear periphery at each stage was indicated.

As a mobile nuclear pore complex component, NUP98 can act both at the nuclear pore complexes and inside the nucleus at sites that are not attached to the nuclear envelope (NE) [8], [17]. Therefore, we wondered if either of the two classes of genes is specifically associated with nuclear pore complexes at the NE. We examined the localization of the group I and group II NUP98 targets by immunofluorescence-fluorescence in situ hybridization (IF-FISH) experiments. We used lamin (LMNB) staining as a marker for the NE, and only counted FISH signals whose center overlaid with the NE (corresponding to <0.5 µm distance from the NE) as ‘periphery’ localization (Figure 7A). We found that the two groups of genes also showed distinct intranuclear localization at the progenitor cell stage. In NeuPCs, group I genes that will become transcriptionally active were localized to the periphery, whereas group II genes that were already expressed at high levels were in the interior of the nucleus (Figure 7B–7D, Figure S11). Upon differentiation into neurons, group I genes moved into the nuclear interior whereas group II genes maintained their interior localization (Figure 7B–7D, Figure S11).

In order to further confirm the association of group I genes with the nuclear pore complexes in NeuPCs, we tested the interaction of these genes with an additional nuclear pore component NUP133 by ChIP-qPCR. NUP133 is a scaffold component of the nuclear pore complexes that associates stably with the nuclear pores at the nuclear periphery [18]. It has not been observed at nuclear pore-free lamina sites or intranuclear sites at endogenous levels. We found that NUP133 bound the group I genes at the nuclear periphery, but not group II genes in the nucleoplasm (Figure S12A). As additional controls, for each group I gene, we selected two neighboring genes for a total of 6 genes (USP16, CLDN17, DCTN16, WRN, KCF7, and PTH2R) and observed no interaction between these genes and NUP98 or NUP133 by ChIP-qPCR (Figure S12A), further supporting the idea that the group I genes interacted with nuclear pores at the nuclear periphery in NeuPCs.

We also examined the intranuclear localization of the 6 neighboring genes (USP16, CLDN17, DCTN16, WRN, KCF7, and PTH2R) to study how far the peripheral localization extended from the group I genes. We found that the 6 genes exhibited large range of percentages of peripheral localization (from 10% to 60%) (Figure S12B). This suggests that NUP98 binding to a given gene at the nuclear periphery could not predict peripheral localization of flanking genes.

### NUP98 regulates neuronal differentiation from neural progenitor cells

Given the association between NUP98 and neural developmental genes, we decided to test if overexpression of full length NUP98 and its dominant negative fragment in neural progenitor cells affected efficiency of neuronal differentiation. We examined the efficiency of neuronal differentiation by measuring the expression levels of markers for differentiated neurons (RBFox3, TUBB3, and Syn1) at the end of 1 month's neuronal differentiation from NeuPCs. We observed that overexpression of full length NUP98 increased expression of those neuronal markers, whereas overexpression of the dominant negative fragment decreased their expression levels (Figure S13). This is consistent with the findings that overexpression of full length NUP98 increased expression of neural developmental genes, whereas overexpression of the fragment reduced expression of such genes (Figure 5). Collectively these results suggest that NUP98 regulates the efficiency of neuronal differentiation from neural progenitor cells. Based on these observations, we conclude that at the neural progenitor stage, there are at least two modes of gene regulation by NUP98, 1) the ‘gene to pore’ model where genes relocate to the nuclear pore at the initial stage of transcriptional induction associated with neurogenesis; and 2) the ‘Nup to gene’ model where NUP98 acts away from the nuclear pore to interact with genes that are highly activated (Figure 7E).

## Discussion

In addition to their well established role in mediating transport across the NE, nuclear pore proteins have been implicated in directly regulating gene expression in organisms as diverse as yeast and *Drosophila* [4], [8]–[13], [16], [17], [20]. However, the functions of Nups during development, especially their roles in gene regulation and in higher organisms such as humans, remain largely unexplored. Here we provide evidence that in human cells, the nuclear pore protein NUP98 binds the nuclear genome in a manner that is tightly linked to differentiation status and developmental gene expression. In embryonic stem cells, NUP98 bound genes include an active subgroup such as genes involved in cell cycle and nucleic acid metabolism regulation and a silent subgroup. In neural progenitor cells, NUP98 shows distinct association with genes activated during neural development, and NUP98 is functionally important for the expression of these genes. In the lung fibroblast IMR90 cells NUP98 mainly interacts with silent chromatin domains. This suggests that besides controlling nucleo-cytoplasmic exchange, NUPs can dynamically interact with the human genome during differentiation, providing an additional layer of genome regulation during development.

From a cell biological point of view, there are at least two modes of developmental gene regulation by NUP98, the ‘on-pore’ regulation and the ‘off-pore’ regulation. Our findings suggest that at least one of the distinctions of the two modes of regulation might be related to the temporal gene expression dynamics of NUP98 targets. Specifically, during the differentiation of human embryonic stem cells along the neural lineage, nuclear pore-tethered NUP98 acts as a short-term anchoring point for certain developmental genes at the beginning stages of transcription induction.

In progenitor cells, anchorage at the nuclear pores could be especially important for genes at the initial stages of developmental induction because for these genes the activation status may not be stable yet and therefore require the microenvironment of the nuclear pores to maintain chromatin decondensation and gene transcriptional status, especially through repeated cell cycles such as in neural progenitor cells (discussed below). On the other hand, for genes that are at later stages of developmental induction, the chromatin is entirely open and thus does not require the nuclear pore-tethering mechanism to maintain transcription. Under such circumstances, the nuclear interior might be a more optimal microenvironment for those genes that supports robust transcription compared to the nuclear pores which are in proximity to the nuclear lamina which can mediate transcriptional repression [45], [46].

The rationale for the involvement of the nuclear pores in developmental gene regulation, especially at the progenitor stage, probably relates to the necessity of re-establishing chromatin organization after nuclear envelope breakdown and reformation in mitosis. During M phase of the cell cycle, chromatin is condensed, transcription activities are largely diminished and most transcription factors are absent from mitotic chromosomes, which composes a window that allows for cell fate reprogramming [47]–[49]. Therefore, in progenitor cells, upon mitosis exit, chromatin has to be decondensed in a manner that faithfully restores the ‘open’ or ‘closed’ states for different chromatin domains to ensure that corresponding developmental genes can be activated or repressed correctly. Nups are prime candidates to regulate transcription re-initiation of developmental genes based on ‘transcriptional memory’ from previous cell cycles because during mitosis exit, Nups are among the first proteins to establish contacts with chromatin and it has been found that proper chromatin decondensation requires the functioning of Nups [50]–[53]. Furthermore, association with Nups in yeast has been shown to convey a ‘gene memory’ function so that genes can be rapidly re-induced for repeated transcription stimulation cycles [12], [54]. Along these lines of evidence, NUP98 in *Drosophila* is involved in the re-initiation of transcription after heat shock [8] and our study has shown that in the cycling human neural progenitor cells NUP98 associates with and regulates expression of neural development genes. Together these observations point to the role of Nups in the rapid and faithful re-initiation of expression of developmental genes after each mitosis cycle.

In the search for DNA sequences that might direct NUP98-chromatin interaction, we identified a conserved DNA binding motif, the GA boxes. This motif is overrepresented in NUP98-binding sequences not only in human cells from our study, but also in *Drosophila* cells from published ChIP-chip and Dam-ID datasets. In *Drosophila*, GA-boxes are recognized by the GAGA factor, which is encoded by the *Trithorax-like* gene and is required for the proper development of the organism [33]. Interestingly, GAGA factor has been related to the yeast factor Rap1 because of their similarities in binding to both repetitive sequences and transcriptionally active genes as well as exhibiting boundary activity [33], and the Rap1 binding site has been identified as the nuclear-pore recognizing DNA motif in yeast [10]. Together these lines of evidence suggest that the DNA recognition activity of Nups or Nup-interacting partners is evolutionarily conserved.

Finally, the involvement of NUP98 in developmental regulation sheds light on its involvement in multiple types of leukemia where it is fused to various transcription regulators [39]. Such oncogenic NUP98-fusion proteins have been shown to promote the self-renewal of hematopoietic progenitor cells and inhibit their differentiation [55]. We found that NUP98 is connected to the regulation of genes implicated in neoplasm formation especially at the progenitor stage. In addition, overexpression of the NUP98 fragment as appeared in the fusion proteins disrupted the expression of endogenous NUP98 targets which, during normal differentiation processes, were activated. Therefore, the misregulation of developmental genes in hematopoietic cells due to genomic fusion of *NUP98* with transcription regulators may be a potential mechanism driving the transformation events in NUP98-fusion protein associated leukemias.

## Materials and Methods

### Ethics statement

Work involving embryonic stem cells was carried out in accordance with the policies set by the Salk Institute.

### Cell culture

Human embryonic stem cell line HUES6 were grown under feeder-free conditions in mTeSR1 medium. HUES6-derived neural progenitor cells were grown in DMEM/F12 supplemented with N2/B27. Early passage IMR90 cells were grown in DMEM, 15% FBS and MEM nonessential amino acids. Culture and differentiation conditions were detailed in Text S1.

### Antibodies

Primary antibodies used include rabbit anti-human NUP98 polyclonal antibody (Cell Signaling 2292; ‘NUP98Ab1’ specified in the experiment), rabbit anti-human NUP98 monoclonal antibody (Cell Signaling 2598), mAb414 (Covance MMS-120R), normal rabbit IgG (Cell Signaling 2729), anti-human Nestin antibody (Chemicon), anti-Sox2 antibody (Chemicon), and rabbit-anti-LMNB antibody (Aviva ARP46357-P050).

### ChIP–Seq and analysis of sequencing data

Cells were fixed in 1% formaldehyde (Polysciences) for 10 min. Fixation was stopped by adding glycine to a final concentration of 125 mM. Fixed cells were lysed and sonicated. DNA was immunoprecipitated, eluted, de-crosslinked, treated with RNase and protease, and purified. Procedures were detailed in Text S1. Library was constructed using Illumina ChIP-Seq DNA sample prep kit and sequencing was done on Illumina GAII. Mapping and peak calling of ChIP-Seq data, annotation of NUP98-binding regions, mapping and expression level analysis of RNA-Seq data, transcription factor motif analysis, gene ontology analysis, positional correlation of ChIP-Seq and RNA-Seq were conducted using the Genomatix software. Peak calling was based on Audic-Claverie algorithm for NGSAnalyzer. Chromosomal views of ChIP-Seq data were generated using Affymetrix Integrated Genome Browser and correlation of NUP98 binding with gene expression levels and histone modification levels was performed using the R package for statistical computing.

### Immunofluorescence–fluorescent in situ hybridization (IF–FISH)

FISH probes were DIG-labelled using the DIG-Nick translation mix for in situ probes (Roche). Cells were fixed, immuno-stained, permeablized, denatured in 50% formamide/2xSSC for 30 min at 80°C, hybridized to DIG-labeled FISH probes overnight at 42°C, stained with anti-DIG antibody (Roche) and Hoechst and mounted. Procedures were detailed in Text S1. Three-dimensional image stacks were recorded with Zeiss LSM710 scanning scope using a 63× objective, 512×512 resolution, 2× averaging and optimal interval (0.31 µm) between stacks in Z-direction and three-dimensional images were reconstructed from the Z-stack images.

### Overexpression and RNAi of *NUPs*

For NUP overexpression, plasmids were electroporated into NeuPCs using rat neural stem cell Nucleofector solution (Lonza Amaxa, VPG-1005) or (in differentiation assays) packaged into lentiviruses that were used subsequently to infect NeuPCs. NUP98 was knocked down by siRNA (oligo sequence: GAG AGA GAT TTA GTT TCC TAA GCA A) in IMR90 cells using Dharmafect 1 siRNA transfection reagent according to the manufacturer's instructions.

## Supporting Information

Figure S1 Validation of ChIP-Seq method. (A) IMR90 cells were stained with two independent anti-human NUP98 antibodies NUP98 Ab1 and NUP98 Ab2 (green), mAb414 (red), and Hoechst (blue). (B) IMR90 cells with scrambled RNAi (scrRi) or NUP98 RNAi (NUP98Ri) were lysed according to the ChIP-Seq protocol and proteins in the lysate were transferred to membrane and blotted with two NUP98 antibodies used for ChIP-Seq (NUP98Ab1 and NUP98Ab2). GAPDH was used as loading control. (C) Immunoprecipitation was performed according to the ChIP-Seq protocol using normal rabbit IgG (IgG) or two NUP98 antibodies (NUP98Ab1, NUP98Ab2) and immunoblotted with NUP98 antibody. (D) Chromosomal view of NUP98 binding regions on chromosome 1 from four independent ChIP-Seq experiments using two NUP98 antibodies under formaldehyde crosslinking condition(NUP98Ab1-F, NUP98Ab2-F) and under formaldehyde-disuccinimidyl glutarate double crosslinking condition (NUP98Ab1-FD, NUP98Ab2-FD). (E) Overlap between NUP98 binding regions from ChIP-Seq experiments using two NUP98 antibodies (NUP98Ab1, NUP98Ab2). (F) Example of peak calling using the Genomatix software. Reads from two NUP98 antibody ChIP-Seq experiments (NUP98Ab1 and NUP98Ab2) and normal rabbit IgG ChIP-Seq experiment (IgG) were shown. Region called as peak by the Genomatix software was indicated by the block in blue (NUP98 Peak). (G) Randomly selected seven ChIP-Seq peaks (T1 from T7) called by Genomatix and two non-NUP98 binding regions (NC1 and NC2) were tested for NUP98 binding by target ChIP-qPCR using independent batch of IMR90 cells and independent lot of NUP98 antibody. Error bars were computed as standard deviation from triplicates. P value was obtained from Student's t-test and comparisons with P value<0.05 indicated with asterisks.
(PNG)

Figure S2 Number of reads from ChIP-Seq experiments. Number of total reads and mappable reads obtained from each ChIP-Seq experiment.
(PNG)

Figure S3 Differentiation of human embryonic stem cells into neural progenitor cells. (A) Scheme showing differentiation of human embryonic stem cells (HESCs) into Embryoid Bodies (EBs), neural rosettes and neural progenitor cells (NeuPCs). The neural progenitor cell cultures are grown as monolayers after neural rosette dissociation. (B) Markers for homogeneous NPC population (Nestin and Sox2) at lower (upper panel) and higher (lower panel) magnification. (C) Quantification of percentage of cells expressing a characteristic neuroprogenitor marker, Nestin. Human embryonic stem cells typically do not express Nestin in contrast to differentiated populations of neural progenitor cells that show homogenous expression of Nestin.
(PNG)

Figure S4 Examples of cell type specific NUP98-binding regions. Reads from NUP98 ChIP-Seq experiments were shown for embryonic stem cells (ESC), neural progenitor cells (NeuPC), neurons (Neuron), and IMR90 cells (IMR90). Peak assigned were indicated in blue. Transcriptional start sites as from the Genomatix database were shown in red. Peaks found in ESCs, NeuPCs and IMR90 cells were shown in (A), (B), and (C), respectively.
(PNG)

Figure S5 Over-represented transcription factor motifs enriched in NUP98-binding regions. (A and B) GA-boxes were over-represented in NUP98-binding genes (A) and NUP98 binding promoters (B) in ESCs and NeuPCs. (C) Over-represented transcription factor motifs in NUP98-binding regions in ESCs and NeuPCs. Transcription factor motifs were ranked by Z-score and motifs with Z-score more than 10 were listed.
(PNG)

Figure S6 Over-represented disease terms enriched in NUP98-binding regions. Disease terms enriched in NUP98 binding genes in NeuPCs by MeSH term analysis.
(PNG)

Figure S7 NUP98 associates with distinct subsets of active and silent genes in embryonic stem cells. (A) Pearson's correlation between pairs of histone modifications for NUP98 binding regions in ESCs. Histone modification levels were calculated from (Lister et al. 2011), GSM605321, and GSM605309. (B, C, and D) For each histone modification type, NUP98 binding genes were ranked by their histone modification levels and top 40% genes were selected for gene ontology analysis. Biological process categories that are uniquely enriched for specific histone modification types were shown in red for active histone marks and in blue for silent histone mark. (E, F, G, and H) Expression levels of NUP98 binding genes that were high in each of the four histone modifications were compared to those of same number of randomly selected genes. P values were obtained by Mann-Whitney U tests. Top and bottom of the boxes in the plot are 25th and 75th percentile, centerline is the 50th, and whiskers extend to 1.5 interquartile range from the upper and lower quantile.
(PNG)

Figure S8 NUP98 or fragment overexpression did not affect expression levels of non-NUP98 binding genes. (A) Fold change in expression levels of non-NUP98 binding genes upon NUP98 overexpression in NeuPCs. Error bars were computed as standard deviation from triplicates. (B) Fold change in expression levels of non-NUP98 binding genes upon NUP98ΔC fragment overexpression in NeuPCs. Error bars were computed as standard deviation from triplicates.
(PNG)

Figure S9 Localization of overexpressed NUP98 and its fragment in neural progenitor cells. (A) Live cell images of neural progenitor cells electroporated with plasmids encoding GFP-tagged full length NUP98 (NUP98-GFP), GFP-tagged NUP98 fragment (NUP98ΔC-GFP), and mock transfected (untreated). GFP channel (left) and phase (right) images were shown. (B) High magnification confocal images of neural progenitor cells fixed after electroporation with plasmids encoding GFP-tagged full length NUP98 (NUP98-GFP) and GFP-tagged NUP98 fragment (NUP98ΔC-GFP). Cells were stained with the nuclear pore marker 414 in red, anti-GFP antibody in green, and Hoechst in blue. In the overlay pictures, a line was drawn across the nuclei and 414 and GFP signals were plotted along the line.
(PNG)

Figure S10 NUP35 overexpression did not affect expression levels of NUP98 binding genes. (A) Fold change in expression levels upon NUP35 overexpression in NeuPCs. Error bars were computed as standard deviation from triplicates. (B) Western blot GAPDH and GFP in NeuPCs with overexpression of GFP-NUP35 or untreated condition as negative control.
(PNG)

Figure S11 Gene localization of two groups of NUP98 targets. Representative 3D IF-FISH images showing the localization of group I genes (NRG1 and MAP2, in A) and group II genes (SOX5 and ERBB4, in B) through development, i.e. in ESC, NeuPC, and Neuron. FISH probes were shown in red, LMNB staining in green, and Hoechst in blue. Each set of images includes the x-y, y-z and x-z planes that cross at the FISH probe signal.
(PNG)

Figure S12 Analysis of neighboring genes of NUP98 targets regarding NUP binding and intranuclear localization. (A) In neural progenitor cells, group I (GRIK1, NRG1, and MAP2) and group II (GPM6B, SOX5, ERBB4) NUP98 targets were tested for NUP98 and NUP133 binding by ChIP-qPCR. For each group I genes, two neighboring genes, one within reported lamin-associated domain (from UCSC genome browser) and one outside of lamin-associated domain, were also analyzed for NUP98 and NUP133 binding (USP16, CLDN17, DCTN16, WRN, KCF7 and PTH2R). GAPDH and ACT genes were used as additional negative control. Error bars were computed as standard deviation from triplicates. (B) Percentage of selected genes localized at the nuclear periphery from IF-FISH experiments in neural progenitor cells.
(PNG)

Figure S13 NUP98 regulates neuronal differentiation. Fold change in expression levels of markers for differentiated neurons in cells overexpressing full length NUP98 (NUP98) or its dominant negative fragment (NUP98ΔC), compared to Untreated control. Neural progenitor cells were mock-infected (Untreated), infected with lentiviruses encoding full length NUP98 (NUP98) or the dominant negative fragment of NUP98 (NUP98ΔC), and differentiated into post-mitotic neurons over a month's course. RNAs were extracted from cells at the end of 1 month's differentiation and RT-qPCR was performed for indicated differentiation markers and GAPDH as a negative control.
(JPG)

Text S1 Inventory of Supplemental Information and Experimental Procedures.
(DOCX)

## References

[1] Alber F, Dokudovskaya S, Veenhoff LM, Zhang W, Kipper J, et al (2007) Determining the architectures of macromolecular assemblies. Nature 450: 683–694. 18046405 10.1038/nature06404

[2] Hetzer MW, Walther TC, Mattaj IW (2005) Pushing the envelope: structure, function, and dynamics of the nuclear periphery. Annu Rev Cell Dev Biol 21: 347–380. 16212499 10.1146/annurev.cellbio.21.090704.151152

[3] Wente SR (2000) Gatekeepers of the nucleus. Science 288: 1374–1377. 10827939 10.1126/science.288.5470.1374

[4] Akhtar A, Gasser SM (2007) The nuclear envelope and transcriptional control. Nat Rev Genet 8: 507–517. 17549064 10.1038/nrg2122

[5] Belmont AS, Zhai Y, Thilenius A (1993) Lamin B distribution and association with peripheral chromatin revealed by optical sectioning and electron microscopy tomography. J Cell Biol 123: 1671–1685. 8276889 10.1083/jcb.123.6.1671 PMC2290888

[6] Schermelleh L, Carlton PM, Haase S, Shao L, Winoto L, et al (2008) Subdiffraction multicolor imaging of the nuclear periphery with 3D structured illumination microscopy. Science 320: 1332–1336. 18535242 10.1126/science.1156947 PMC2916659

[7] Blobel G (1985) Gene gating: a hypothesis. Proc Natl Acad Sci U S A 82: 8527–8529. 3866238 10.1073/pnas.82.24.8527 PMC390949

[8] Capelson M, Liang Y, Schulte R, Mair W, Wagner U, et al (2010) Chromatin-bound nuclear pore components regulate gene expression in higher eukaryotes. Cell 140: 372–383. 20144761 10.1016/j.cell.2009.12.054 PMC2821818

[9] Casolari JM, Brown CR, Drubin DA, Rando OJ, Silver PA (2005) Developmentally induced changes in transcriptional program alter spatial organization across chromosomes. Genes Dev 19: 1188–1198. 15905407 10.1101/gad.1307205 PMC1132005

[10] Casolari JM, Brown CR, Komili S, West J, Hieronymus H, et al (2004) Genome-wide localization of the nuclear transport machinery couples transcriptional status and nuclear organization. Cell 117: 427–439. 15137937 10.1016/s0092-8674(04)00448-9

[11] Taddei A, Van Houwe G, Hediger F, Kalck V, Cubizolles F, et al (2006) Nuclear pore association confers optimal expression levels for an inducible yeast gene. Nature 441: 774–778. 16760983 10.1038/nature04845

[12] Tan-Wong SM, Wijayatilake HD, Proudfoot NJ (2009) Gene loops function to maintain transcriptional memory through interaction with the nuclear pore complex. Genes Dev 23: 2610–2624. 19933151 10.1101/gad.1823209 PMC2779764

[13] Vaquerizas JM, Suyama R, Kind J, Miura K, Luscombe NM, et al (2010) Nuclear pore proteins nup153 and megator define transcriptionally active regions in the Drosophila genome. PLoS Genet 6: e1000846 doi:10.1371/journal.pgen.1000846 20174442 10.1371/journal.pgen.1000846 PMC2820533

[14] Brickner JH, Walter P (2004) Gene recruitment of the activated INO1 locus to the nuclear membrane. PLoS Biol 2: e342 doi:10.1371/journal.pbio.0020342 15455074 10.1371/journal.pbio.0020342 PMC519002

[15] Dilworth DJ, Tackett AJ, Rogers RS, Yi EC, Christmas RH, et al (2005) The mobile nucleoporin Nup2p and chromatin-bound Prp20p function in endogenous NPC-mediated transcriptional control. J Cell Biol 171: 955–965. 16365162 10.1083/jcb.200509061 PMC2171315

[16] Ishii K, Arib G, Lin C, Van Houwe G, Laemmli UK (2002) Chromatin boundaries in budding yeast: the nuclear pore connection. Cell 109: 551–562. 12062099 10.1016/s0092-8674(02)00756-0

[17] Kalverda B, Pickersgill H, Shloma VV, Fornerod M (2010) Nucleoporins directly stimulate expression of developmental and cell-cycle genes inside the nucleoplasm. Cell 140: 360–371. 20144760 10.1016/j.cell.2010.01.011

[18] Griffis ER, Xu S, Powers MA (2003) Nup98 localizes to both nuclear and cytoplasmic sides of the nuclear pore and binds to two distinct nucleoporin subcomplexes. Mol Biol Cell 14: 600–610. 12589057 10.1091/mbc.E02-09-0582 PMC149995

[19] Kehat I, Accornero F, Aronow BJ, Molkentin JD (2011) Modulation of chromatin position and gene expression by HDAC4 interaction with nucleoporins. J Cell Biol 193: 21–29. 21464227 10.1083/jcb.201101046 PMC3082185

[20] Brown CR, Kennedy CJ, Delmar VA, Forbes DJ, Silver PA (2008) Global histone acetylation induces functional genomic reorganization at mammalian nuclear pore complexes. Genes Dev 22: 627–639. 18316479 10.1101/gad.1632708 PMC2259032

[21] Schneider R, Grosschedl R (2007) Dynamics and interplay of nuclear architecture, genome organization, and gene expression. Genes Dev 21: 3027–3043. 18056419 10.1101/gad.1604607

[22] Mattout A, Meshorer E (2010) Chromatin plasticity and genome organization in pluripotent embryonic stem cells. Curr Opin Cell Biol 22: 334–341. 20226651 10.1016/j.ceb.2010.02.001

[23] Meshorer E, Misteli T (2006) Chromatin in pluripotent embryonic stem cells and differentiation. Nat Rev Mol Cell Biol 7: 540–546. 16723974 10.1038/nrm1938

[24] Brown KE, Amoils S, Horn JM, Buckle VJ, Higgs DR, et al (2001) Expression of alpha- and beta-globin genes occurs within different nuclear domains in haemopoietic cells. Nat Cell Biol 3: 602–606. 11389446 10.1038/35078577

[25] Kosak ST, Skok JA, Medina KL, Riblet R, Le Beau MM, et al (2002) Subnuclear compartmentalization of immunoglobulin loci during lymphocyte development. Science 296: 158–162. 11935030 10.1126/science.1068768

[26] Williams RR, Azuara V, Perry P, Sauer S, Dvorkina M, et al (2006) Neural induction promotes large-scale chromatin reorganisation of the Mash1 locus. J Cell Sci 119: 132–140. 16371653 10.1242/jcs.02727

[27] Wiblin AE, Cui W, Clark AJ, Bickmore WA (2005) Distinctive nuclear organisation of centromeres and regions involved in pluripotency in human embryonic stem cells. J Cell Sci 118: 3861–3868. 16105879 10.1242/jcs.02500

[28] Meshorer E, Yellajoshula D, George E, Scambler PJ, Brown DT, et al (2006) Hyperdynamic plasticity of chromatin proteins in pluripotent embryonic stem cells. Dev Cell 10: 105–116. 16399082 10.1016/j.devcel.2005.10.017 PMC1868458

[29] Efroni S, Duttagupta R, Cheng J, Dehghani H, Hoeppner DJ, et al (2008) Global transcription in pluripotent embryonic stem cells. Cell Stem Cell 2: 437–447. 18462694 10.1016/j.stem.2008.03.021 PMC2435228

[30] Meister P, Towbin BD, Pike BL, Ponti A, Gasser SM (2010) The spatial dynamics of tissue-specific promoters during C. elegans development. Genes Dev 24: 766–782. 20395364 10.1101/gad.559610 PMC2854392

[31] Capelson M, Hetzer MW (2009) The role of nuclear pores in gene regulation, development and disease. EMBO Rep 10: 697–705. 19543230 10.1038/embor.2009.147 PMC2727434

[32] Griffis ER, Altan N, Lippincott-Schwartz J, Powers MA (2002) Nup98 is a mobile nucleoporin with transcription-dependent dynamics. Mol Biol Cell 13: 1282–1297. 11950939 10.1091/mbc.01-11-0538 PMC102269

[33] Granok H, Leibovitch BA, Shaffer CD, Elgin SC (1995) Chromatin. Ga-ga over GAGA factor. Curr Biol 5: 238–241. 7780729 10.1016/s0960-9822(95)00048-0

[34] Ohtsuki S, Levine M (1998) GAGA mediates the enhancer blocking activity of the eve promoter in the Drosophila embryo. Genes Dev 12: 3325–3330. 9808619 10.1101/gad.12.21.3325 PMC317233

[35] Wallace JA, Felsenfeld G (2007) We gather together: insulators and genome organization. Curr Opin Genet Dev 17: 400–407. 17913488 10.1016/j.gde.2007.08.005 PMC2215060

[36] Krull S, Dorries J, Boysen B, Reidenbach S, Magnius L, et al (2010) Protein Tpr is required for establishing nuclear pore-associated zones of heterochromatin exclusion. EMBO J 29: 1659–1673. 20407419 10.1038/emboj.2010.54 PMC2876962

[37] Huggenvik JI, Michelson RJ, Collard MW, Ziemba AJ, Gurley P, et al (1998) Characterization of a nuclear deformed epidermal autoregulatory factor-1 (DEAF-1)-related (NUDR) transcriptional regulator protein. Mol Endocrinol 12: 1619–1639. 9773984 10.1210/mend.12.10.0181

[38] Zhong S, Salomoni P, Pandolfi PP (2000) The transcriptional role of PML and the nuclear body. Nat Cell Biol 2: E85–90. 10806494 10.1038/35010583

[39] Moore MA, Chung KY, Plasilova M, Schuringa JJ, Shieh JH, et al (2007) NUP98 dysregulation in myeloid leukemogenesis. Ann N Y Acad Sci 1106: 114–142. 17442773 10.1196/annals.1392.019

[40] Wang J, Rao S, Chu J, Shen X, Levasseur DN, et al (2006) A protein interaction network for pluripotency of embryonic stem cells. Nature 444: 364–368. 17093407 10.1038/nature05284

[41] Xu RH, Sampsell-Barron TL, Gu F, Root S, Peck RM, et al (2008) NANOG is a direct target of TGFbeta/activin-mediated SMAD signaling in human ESCs. Cell Stem Cell 3: 196–206. 18682241 10.1016/j.stem.2008.07.001 PMC2758041

[42] Lister R, Pelizzola M, Kida YS, Hawkins RD, Nery JR, et al (2011) Hotspots of aberrant epigenomic reprogramming in human induced pluripotent stem cells. Nature 471: 68–73. 21289626 10.1038/nature09798 PMC3100360

[43] Wu JQ, Habegger L, Noisa P, Szekely A, Qiu C, et al (2010) Dynamic transcriptomes during neural differentiation of human embryonic stem cells revealed by short, long, and paired-end sequencing. Proc Natl Acad Sci U S A 107: 5254–5259. 20194744 10.1073/pnas.0914114107 PMC2841935

[44] Kouzarides T (2007) Chromatin modifications and their function. Cell 128: 693–705. 17320507 10.1016/j.cell.2007.02.005

[45] Reddy KL, Zullo JM, Bertolino E, Singh H (2008) Transcriptional repression mediated by repositioning of genes to the nuclear lamina. Nature 452: 243–247. 18272965 10.1038/nature06727

[46] Lee DC, Welton KL, Smith ED, Kennedy BK (2009) A-type nuclear lamins act as transcriptional repressors when targeted to promoters. Exp Cell Res 315: 996–1007. 19272320 10.1016/j.yexcr.2009.01.003 PMC2746445

[47] Martinez-Balbas MA, Dey A, Rabindran SK, Ozato K, Wu C (1995) Displacement of sequence-specific transcription factors from mitotic chromatin. Cell 83: 29–38. 7553870 10.1016/0092-8674(95)90231-7

[48] Taylor JH (1960) Nucleic acid synthesis in relation to the cell division cycle. Ann N Y Acad Sci 90: 409–421. 13775619 10.1111/j.1749-6632.1960.tb23259.x

[49] Egli D, Birkhoff G, Eggan K (2008) Mediators of reprogramming: transcription factors and transitions through mitosis. Nat Rev Mol Cell Biol 9: 505–516. 18568039 10.1038/nrm2439 PMC7250051

[50] Franz C, Walczak R, Yavuz S, Santarella R, Gentzel M, et al (2007) MEL-28/ELYS is required for the recruitment of nucleoporins to chromatin and postmitotic nuclear pore complex assembly. EMBO Rep 8: 165–172. 17235358 10.1038/sj.embor.7400889 PMC1796766

[51] Rasala BA, Orjalo AV, Shen Z, Briggs S, Forbes DJ (2006) ELYS is a dual nucleoporin/kinetochore protein required for nuclear pore assembly and proper cell division. Proc Natl Acad Sci U S A 103: 17801–17806. 17098863 10.1073/pnas.0608484103 PMC1635652

[52] Walther TC, Alves A, Pickersgill H, Loiodice I, Hetzer M, et al (2003) The conserved Nup107–160 complex is critical for nuclear pore complex assembly. Cell 113: 195–206. 12705868 10.1016/s0092-8674(03)00235-6

[53] Benavente R, Dabauvalle MC, Scheer U, Chaly N (1989) Functional role of newly formed pore complexes in postmitotic nuclear reorganization. Chromosoma 98: 233–241. 2692995 10.1007/BF00327308

[54] Light WH, Brickner DG, Brand VR, Brickner JH (2010) Interaction of a DNA zip code with the nuclear pore complex promotes H2A.Z incorporation and INO1 transcriptional memory. Mol Cell 40: 112–125. 20932479 10.1016/j.molcel.2010.09.007 PMC2953765

[55] Gough SM, Slape CI, Aplan PD (2011) NUP98 gene fusions and hematopoietic malignancies: common themes and new biological insights. Blood 118: 6247–6257. 21948299 10.1182/blood-2011-07-328880 PMC3236115

