# Supplementary figures and images for: Dynamic Association of NUP98 with the Human Genome
Source: PLoS Genet. 2013 Feb 28;9(2):e1003308. doi: 10.1371/journal.pgen.1003308 (PMC3585015; doi:10.1371/journal.pgen.1003308)

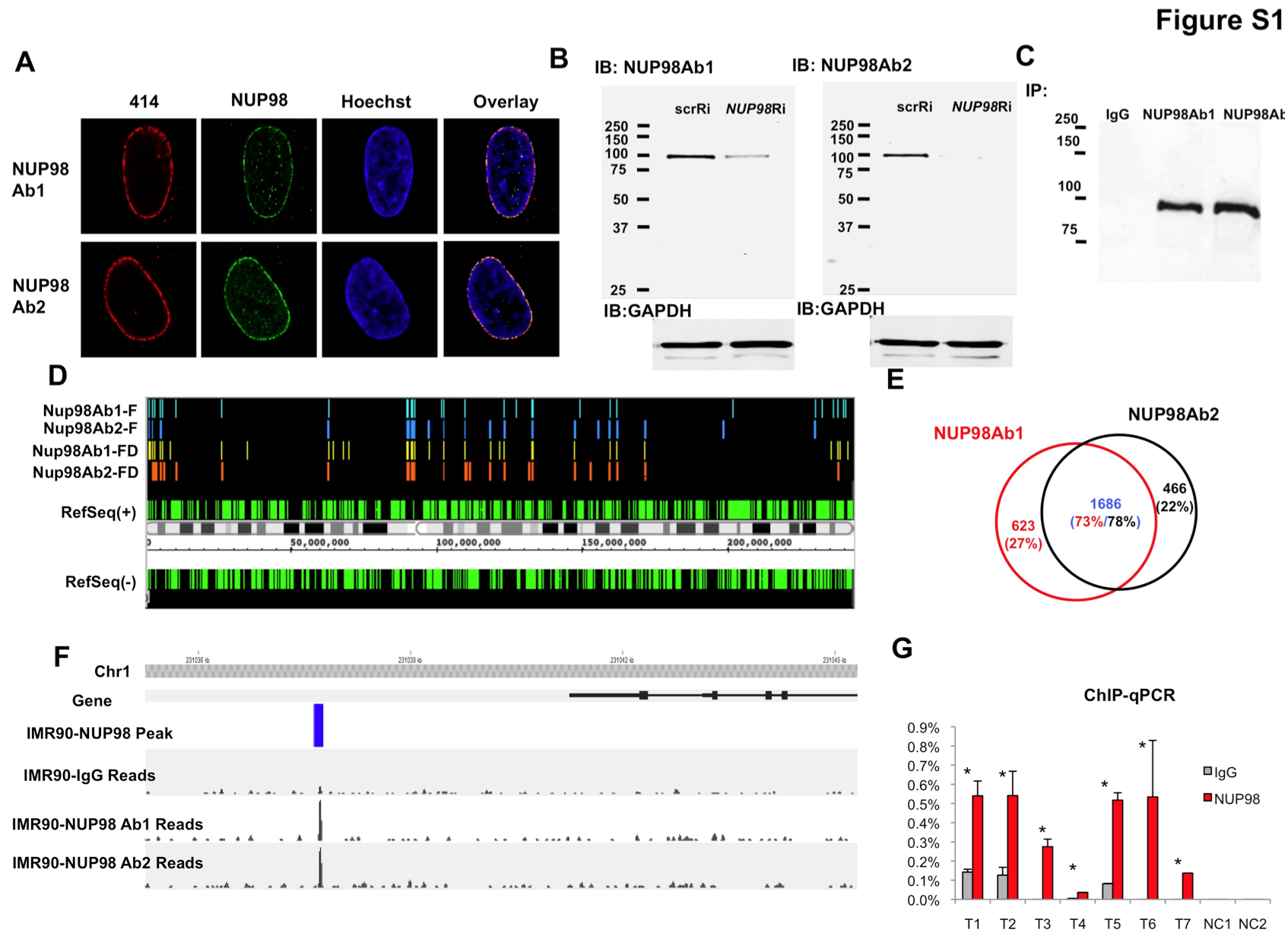

Supplement: Figure S1 — Validation of ChIP-Seq method. (A) IMR90 cells were stained with two independent anti-human NUP98 antibodies NUP98 Ab1 and NUP98 Ab2 (green), mAb414 (red), and Hoechst (blue). (B) IMR90 cells with scrambled RNAi (scrRi) or NUP98 RNAi (NUP98Ri) were lysed according to the ChIP-Seq protocol and proteins in the lysate were transferred to membrane and blotted with two NUP98 antibodies used for ChIP-Seq (NUP98Ab1 and NUP98Ab2). GAPDH was used as loading control. (C) Immunoprecipitation was performed according to the ChIP-Seq protocol using normal rabbit IgG (IgG) or two NUP98 antibodies (NUP98Ab1, NUP98Ab2) and immunoblotted with NUP98 antibody. (D) Chromosomal view of NUP98 binding regions on chromosome 1 from four independent ChIP-Seq experiments using two NUP98 antibodies under formaldehyde crosslinking condition(NUP98Ab1-F, NUP98Ab2-F) and under formaldehyde-disuccinimidyl glutarate double crosslinking condition (NUP98Ab1-FD, NUP98Ab2-FD). (E) Overlap between NUP98 binding regions from ChIP-Seq experiments using two NUP98 antibodies (NUP98Ab1, NUP98Ab2). (F) Example of peak calling using the Genomatix software. Reads from two NUP98 antibody ChIP-Seq experiments (NUP98Ab1 and NUP98Ab2) and normal rabbit IgG ChIP-Seq experiment (IgG) were shown. Region called as peak by the Genomatix software was indicated by the block in blue (NUP98 Peak). (G) Randomly selected seven ChIP-Seq peaks (T1 from T7) called by Genomatix and two non-NUP98 binding regions (NC1 and NC2) were tested for NUP98 binding by target ChIP-qPCR using independent batch of IMR90 cells and independent lot of NUP98 antibody. Error bars were computed as standard deviation from triplicates. P value was obtained from Student's t-test and comparisons with P value<0.05 indicated with asterisks. (PNG) [file pgen.1003308.s001.png]

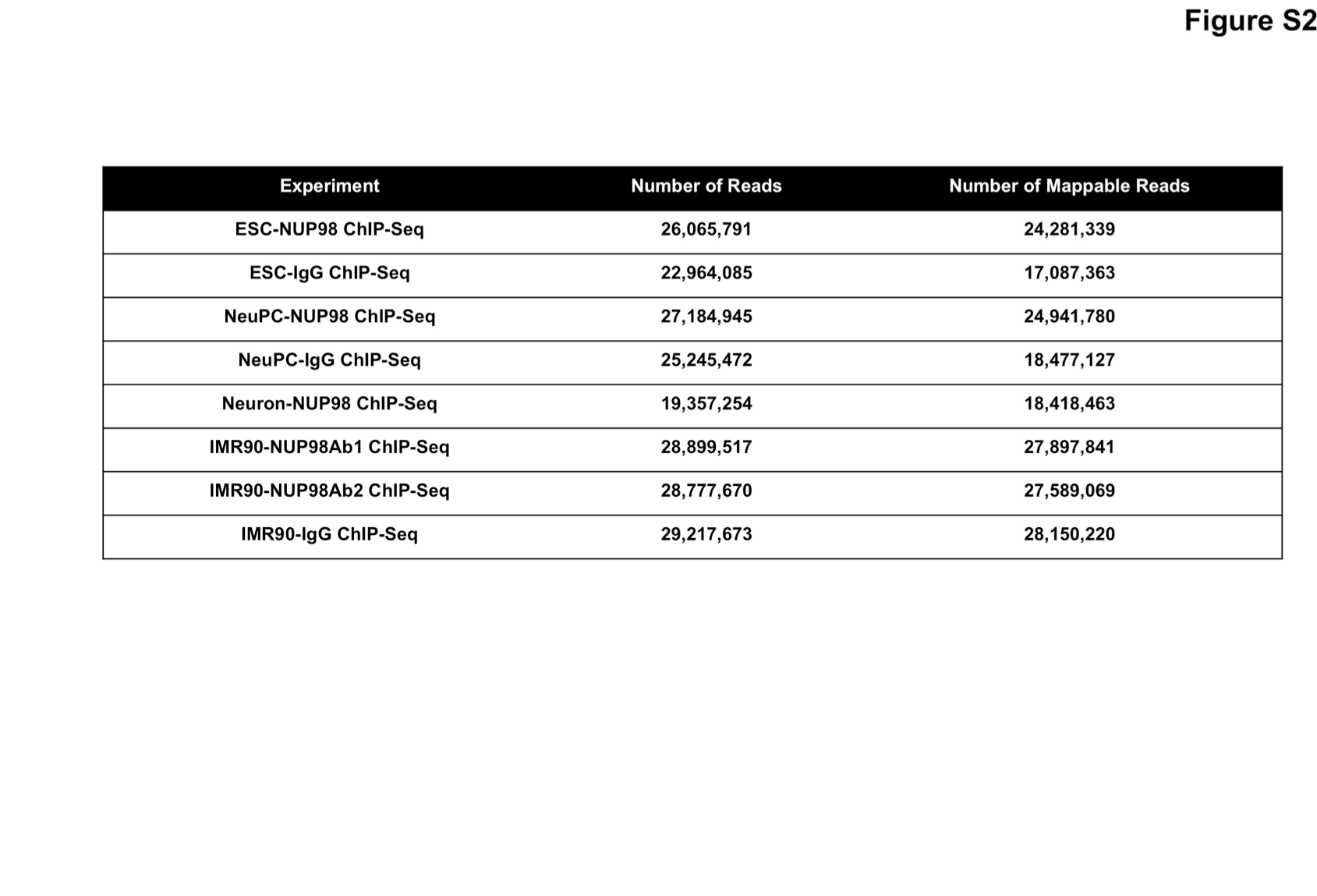

Supplement: Figure S2 — Number of reads from ChIP-Seq experiments. Number of total reads and mappable reads obtained from each ChIP-Seq experiment. (PNG) [file pgen.1003308.s002.png]

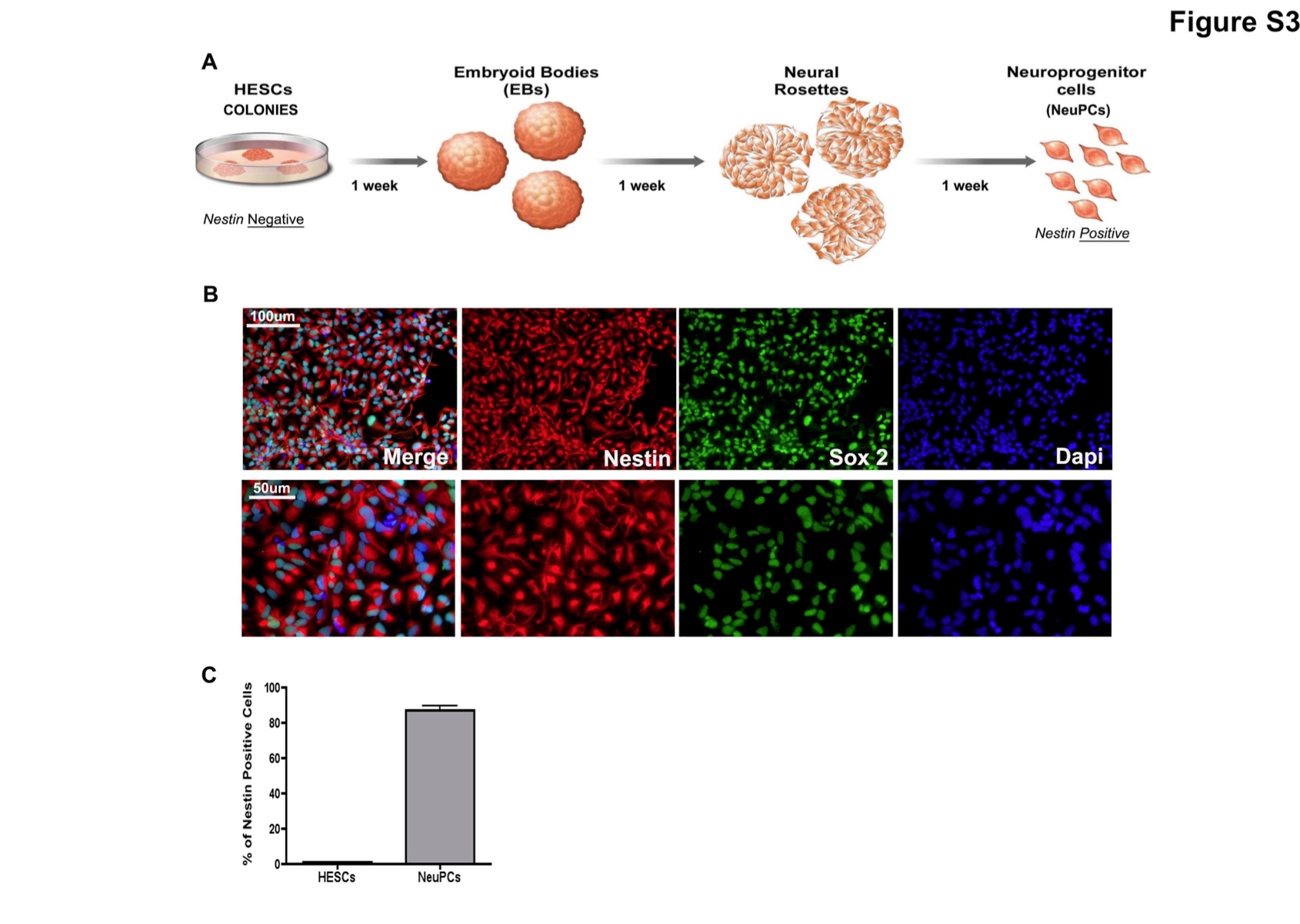

Supplement: Figure S3 — Differentiation of human embryonic stem cells into neural progenitor cells. (A) Scheme showing differentiation of human embryonic stem cells (HESCs) into Embryoid Bodies (EBs), neural rosettes and neural progenitor cells (NeuPCs). The neural progenitor cell cultures are grown as monolayers after neural rosette dissociation. (B) Markers for homogeneous NPC population (Nestin and Sox2) at lower (upper panel) and higher (lower panel) magnification. (C) Quantification of percentage of cells expressing a characteristic neuroprogenitor marker, Nestin. Human embryonic stem cells typically do not express Nestin in contrast to differentiated populations of neural progenitor cells that show homogenous expression of Nestin. (PNG) [file pgen.1003308.s003.png]

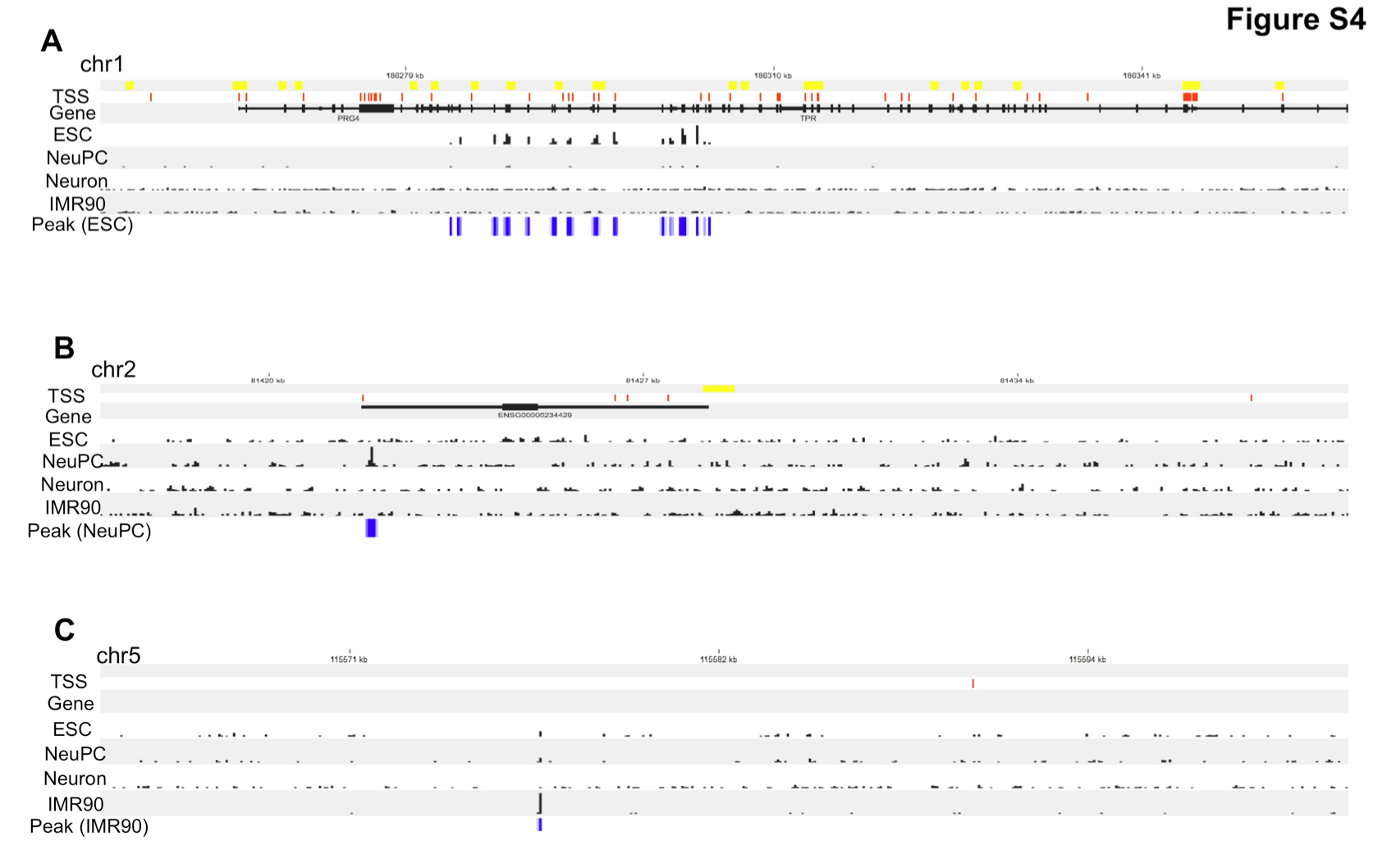

Supplement: Figure S4 — Examples of cell type specific NUP98-binding regions. Reads from NUP98 ChIP-Seq experiments were shown for embryonic stem cells (ESC), neural progenitor cells (NeuPC), neurons (Neuron), and IMR90 cells (IMR90). Peak assigned were indicated in blue. Transcriptional start sites as from the Genomatix database were shown in red. Peaks found in ESCs, NeuPCs and IMR90 cells were shown in (A), (B), and (C), respectively. (PNG) [file pgen.1003308.s004.png]

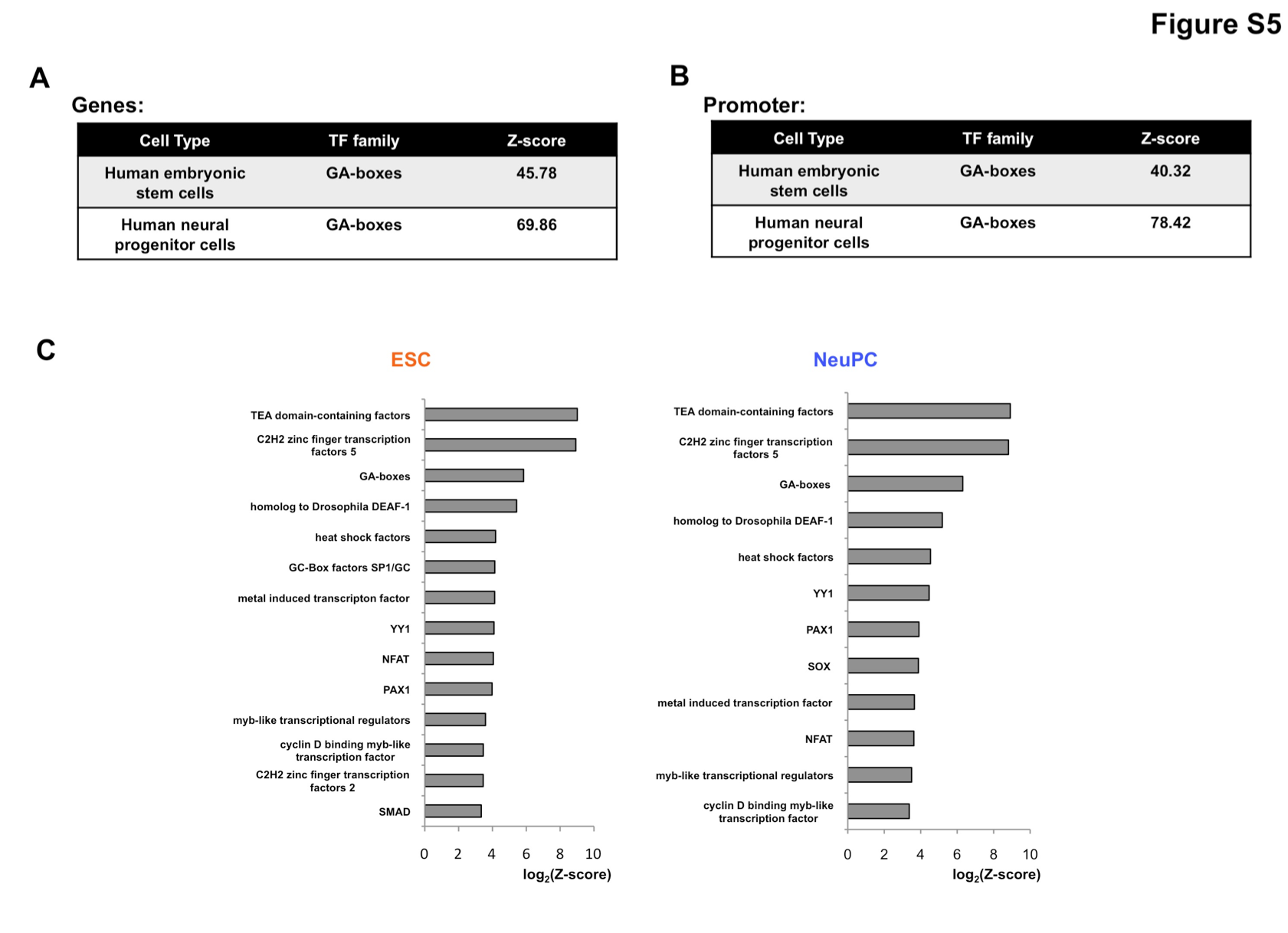

Supplement: Figure S5 — Over-represented transcription factor motifs enriched in NUP98-binding regions. (A and B) GA-boxes were over-represented in NUP98-binding genes (A) and NUP98 binding promoters (B) in ESCs and NeuPCs. (C) Over-represented transcription factor motifs in NUP98-binding regions in ESCs and NeuPCs. Transcription factor motifs were ranked by Z-score and motifs with Z-score more than 10 were listed. (PNG) [file pgen.1003308.s005.png]

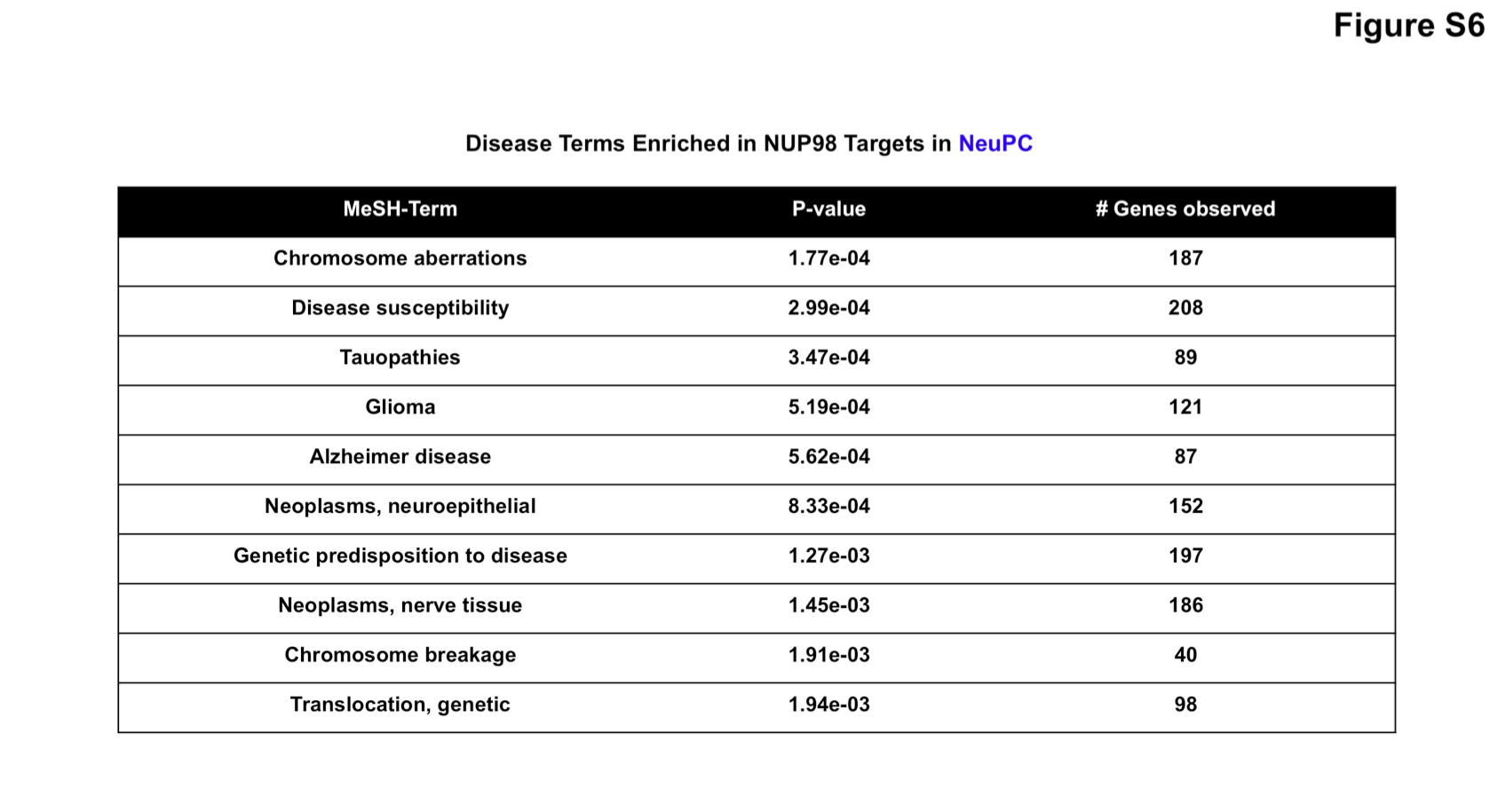

Supplement: Figure S6 — Over-represented disease terms enriched in NUP98-binding regions. Disease terms enriched in NUP98 binding genes in NeuPCs by MeSH term analysis. (PNG) [file pgen.1003308.s006.png]

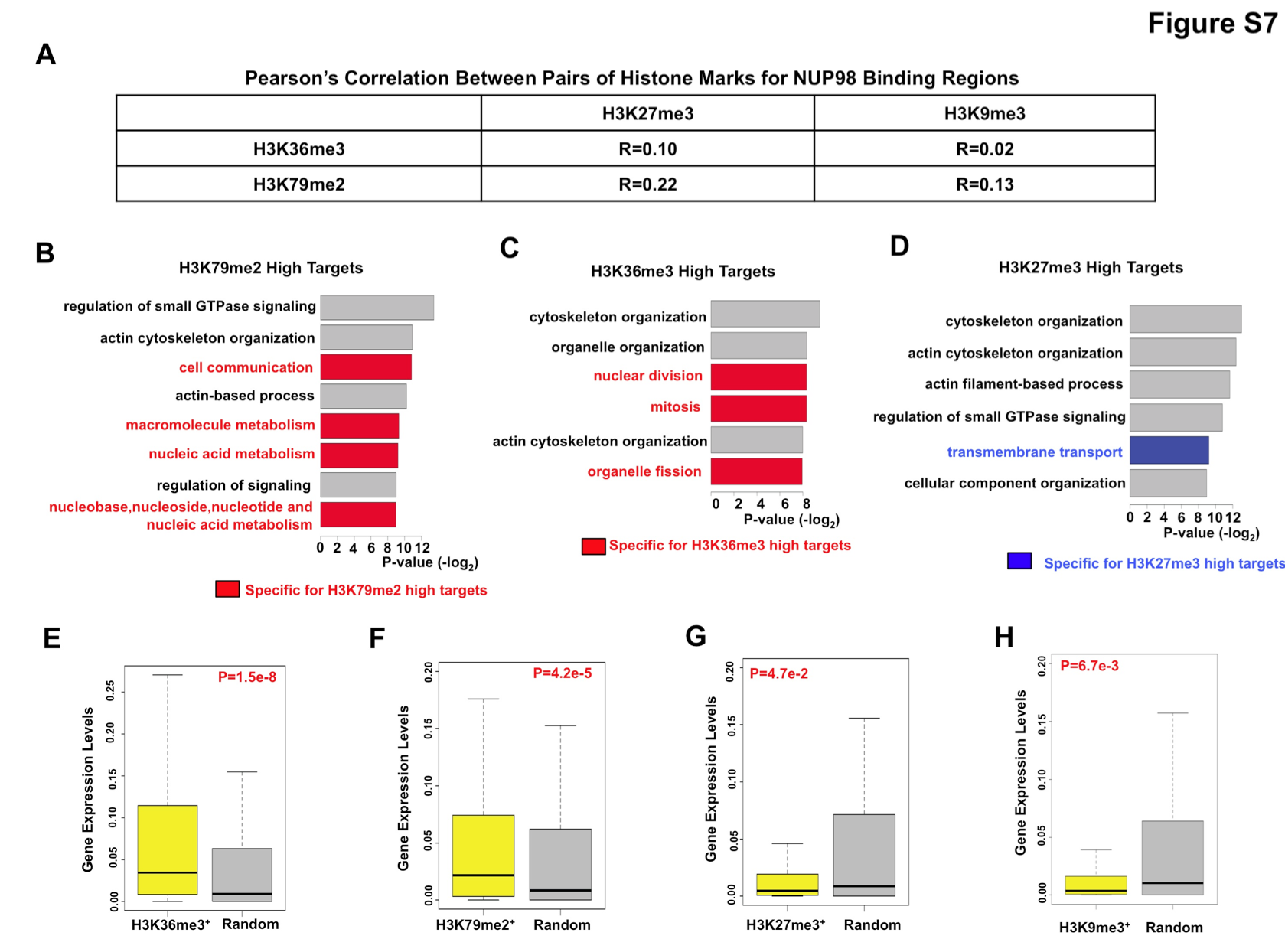

Supplement: Figure S7 — NUP98 associates with distinct subsets of active and silent genes in embryonic stem cells. (A) Pearson's correlation between pairs of histone modifications for NUP98 binding regions in ESCs. Histone modification levels were calculated from (Lister et al. 2011), GSM605321, and GSM605309. (B, C, and D) For each histone modification type, NUP98 binding genes were ranked by their histone modification levels and top 40% genes were selected for gene ontology analysis. Biological process categories that are uniquely enriched for specific histone modification types were shown in red for active histone marks and in blue for silent histone mark. (E, F, G, and H) Expression levels of NUP98 binding genes that were high in each of the four histone modifications were compared to those of same number of randomly selected genes. P values were obtained by Mann-Whitney U tests. Top and bottom of the boxes in the plot are 25th and 75th percentile, centerline is the 50th, and whiskers extend to 1.5 interquartile range from the upper and lower quantile. (PNG) [file pgen.1003308.s007.png]

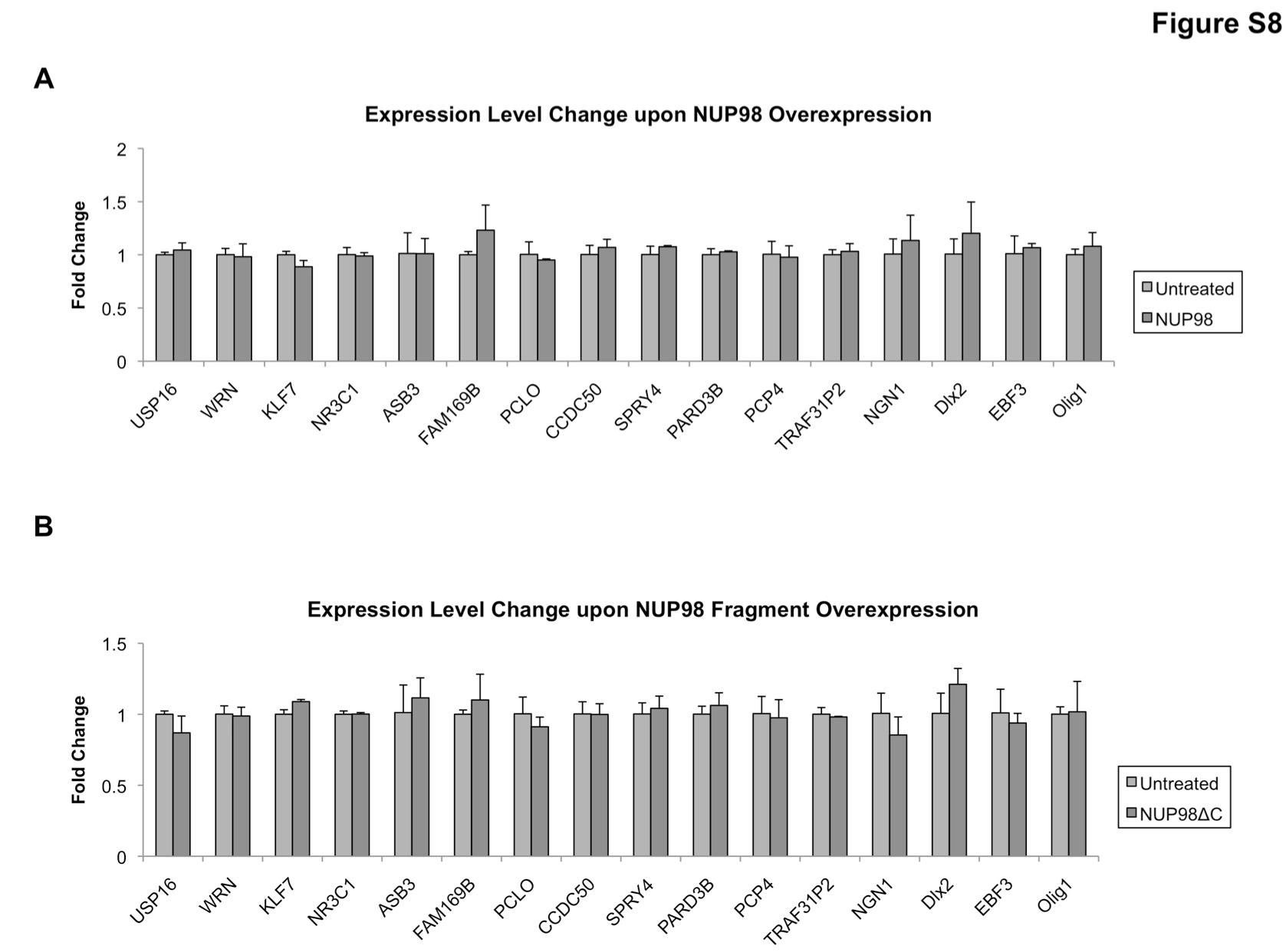

Supplement: Figure S8 — NUP98 or fragment overexpression did not affect expression levels of non-NUP98 binding genes. (A) Fold change in expression levels of non-NUP98 binding genes upon NUP98 overexpression in NeuPCs. Error bars were computed as standard deviation from triplicates. (B) Fold change in expression levels of non-NUP98 binding genes upon NUP98ΔC fragment overexpression in NeuPCs. Error bars were computed as standard deviation from triplicates. (PNG) [file pgen.1003308.s008.png]

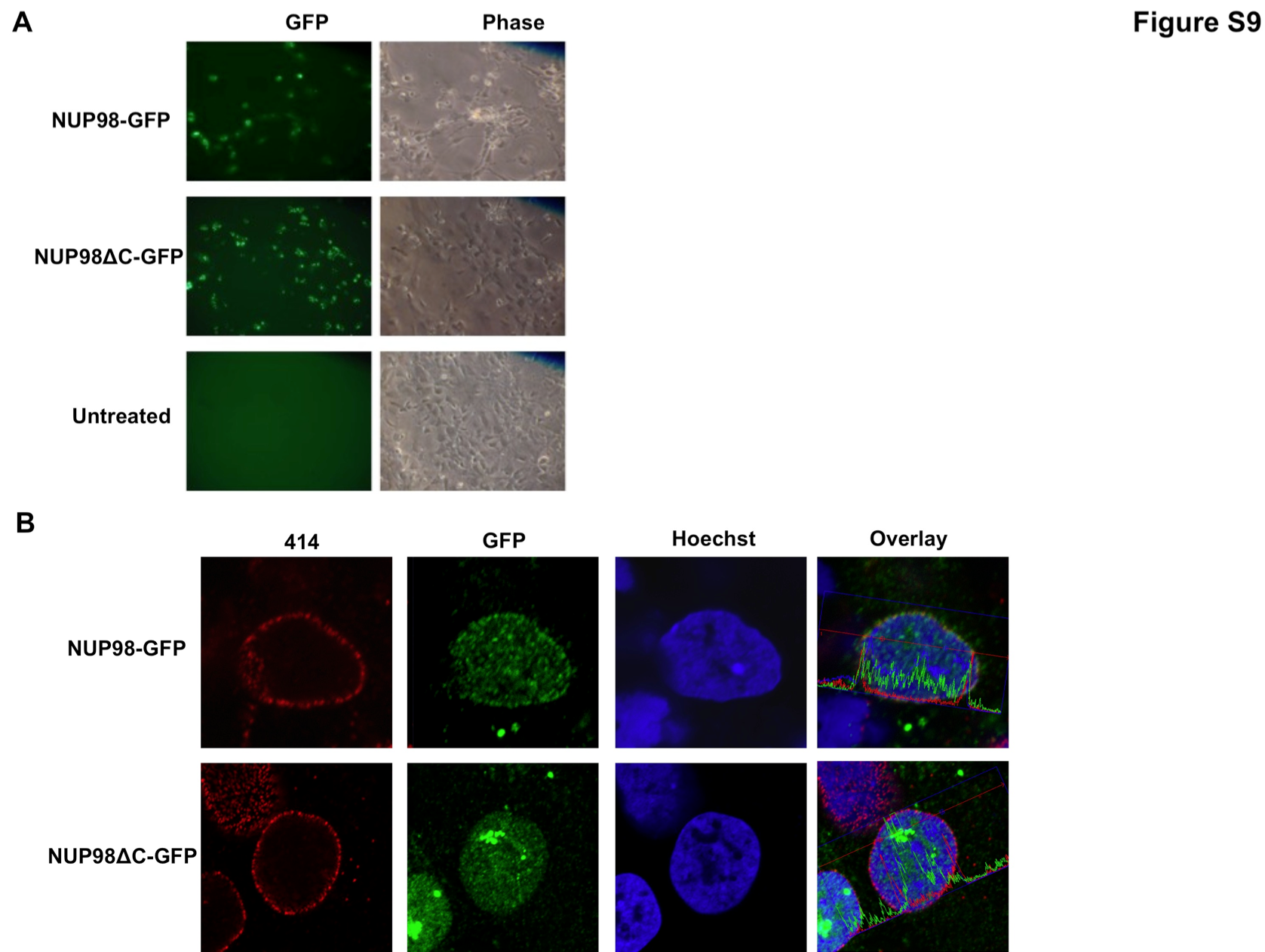

Supplement: Figure S9 — Localization of overexpressed NUP98 and its fragment in neural progenitor cells. (A) Live cell images of neural progenitor cells electroporated with plasmids encoding GFP-tagged full length NUP98 (NUP98-GFP), GFP-tagged NUP98 fragment (NUP98ΔC-GFP), and mock transfected (untreated). GFP channel (left) and phase (right) images were shown. (B) High magnification confocal images of neural progenitor cells fixed after electroporation with plasmids encoding GFP-tagged full length NUP98 (NUP98-GFP) and GFP-tagged NUP98 fragment (NUP98ΔC-GFP). Cells were stained with the nuclear pore marker 414 in red, anti-GFP antibody in green, and Hoechst in blue. In the overlay pictures, a line was drawn across the nuclei and 414 and GFP signals were plotted along the line. (PNG) [file pgen.1003308.s009.png]

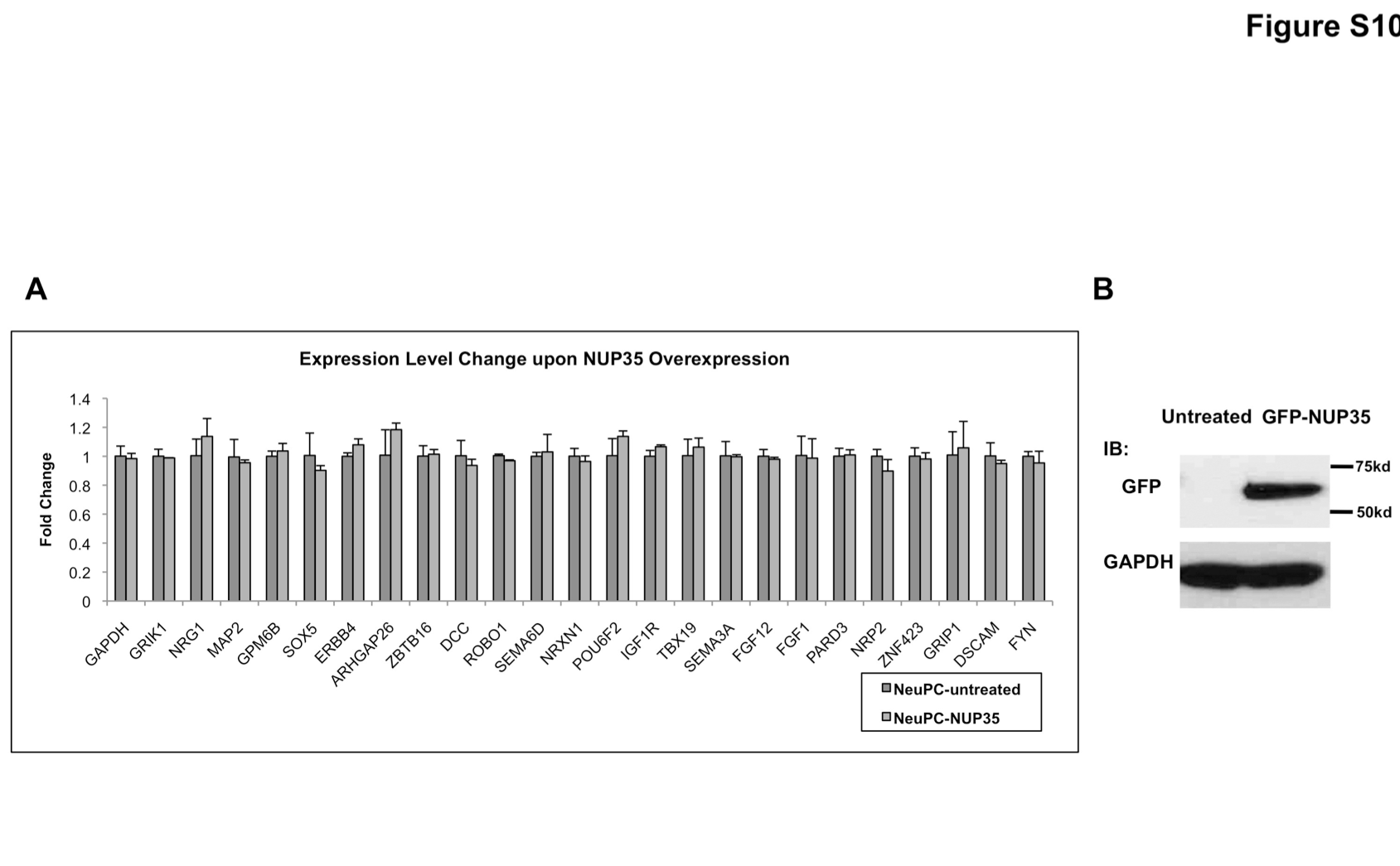

Supplement: Figure S10 — NUP35 overexpression did not affect expression levels of NUP98 binding genes. (A) Fold change in expression levels upon NUP35 overexpression in NeuPCs. Error bars were computed as standard deviation from triplicates. (B) Western blot GAPDH and GFP in NeuPCs with overexpression of GFP-NUP35 or untreated condition as negative control. (PNG) [file pgen.1003308.s010.png]

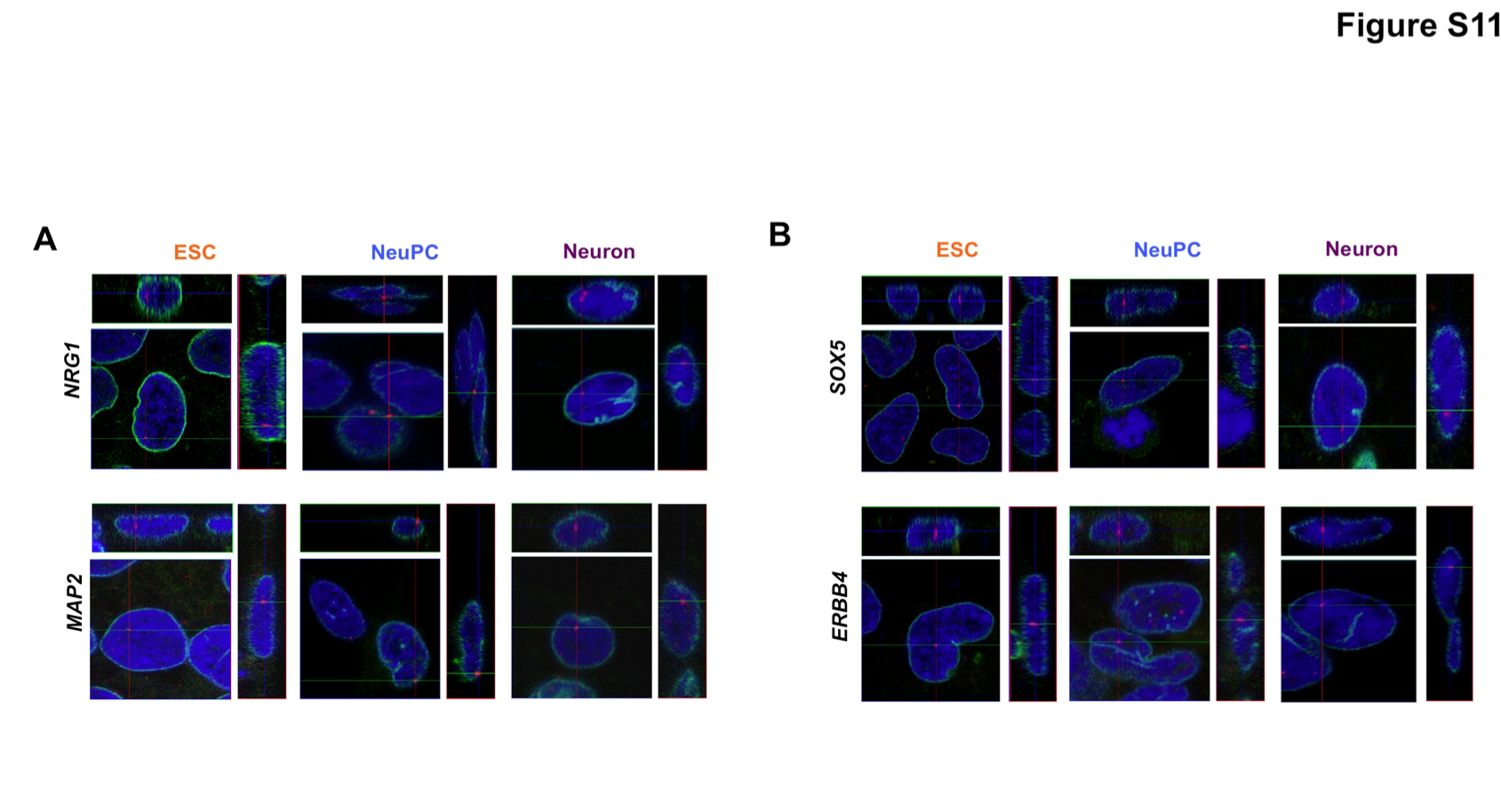

Supplement: Figure S11 — Gene localization of two groups of NUP98 targets. Representative 3D IF-FISH images showing the localization of group I genes (NRG1 and MAP2, in A) and group II genes (SOX5 and ERBB4, in B) through development, i.e. in ESC, NeuPC, and Neuron. FISH probes were shown in red, LMNB staining in green, and Hoechst in blue. Each set of images includes the x-y, y-z and x-z planes that cross at the FISH probe signal. (PNG) [file pgen.1003308.s011.png]

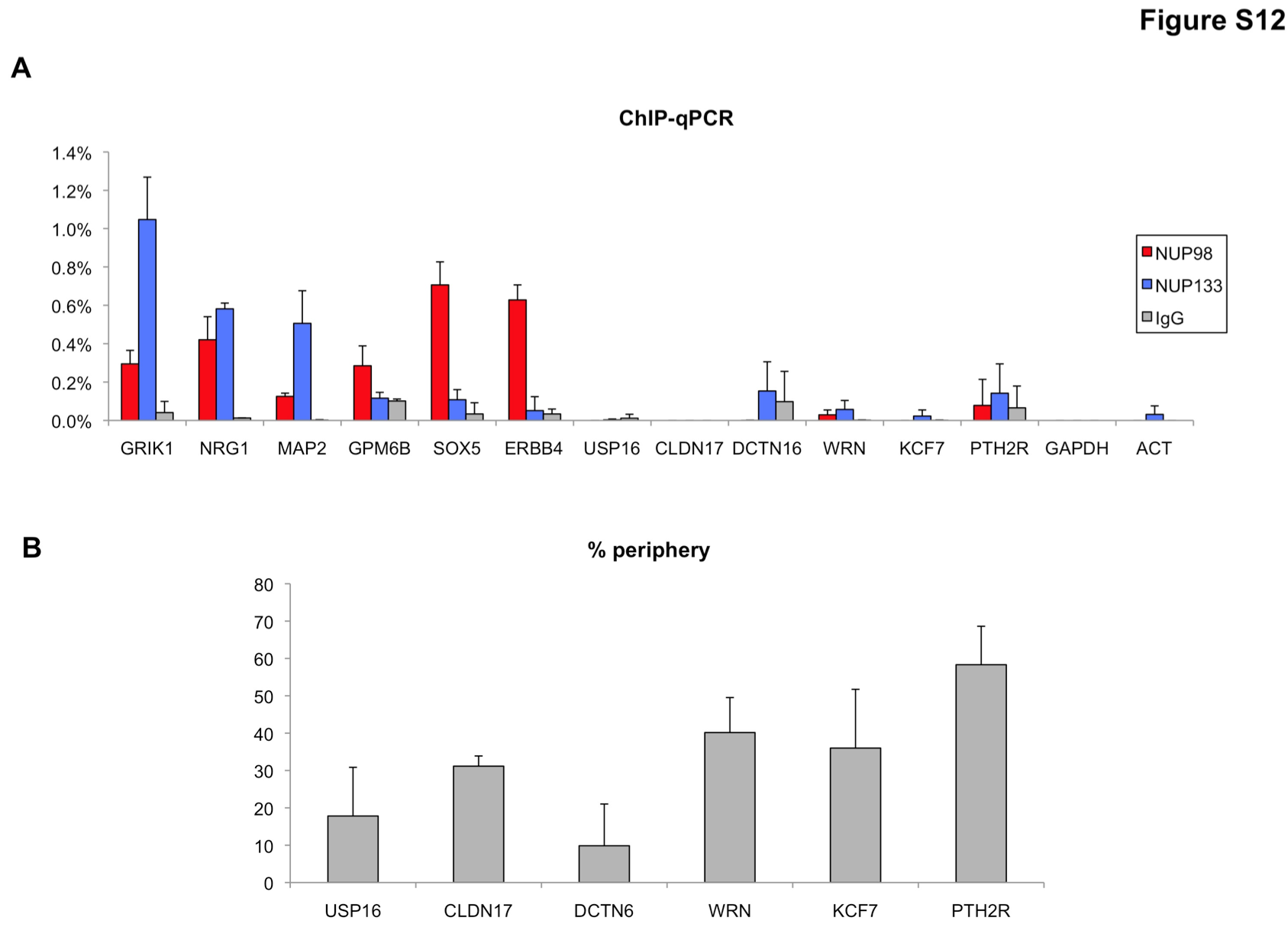

Supplement: Figure S12 — Analysis of neighboring genes of NUP98 targets regarding NUP binding and intranuclear localization. (A) In neural progenitor cells, group I (GRIK1, NRG1, and MAP2) and group II (GPM6B, SOX5, ERBB4) NUP98 targets were tested for NUP98 and NUP133 binding by ChIP-qPCR. For each group I genes, two neighboring genes, one within reported lamin-associated domain (from UCSC genome browser) and one outside of lamin-associated domain, were also analyzed for NUP98 and NUP133 binding (USP16, CLDN17, DCTN16, WRN, KCF7 and PTH2R). GAPDH and ACT genes were used as additional negative control. Error bars were computed as standard deviation from triplicates. (B) Percentage of selected genes localized at the nuclear periphery from IF-FISH experiments in neural progenitor cells. (PNG) [file pgen.1003308.s012.png]

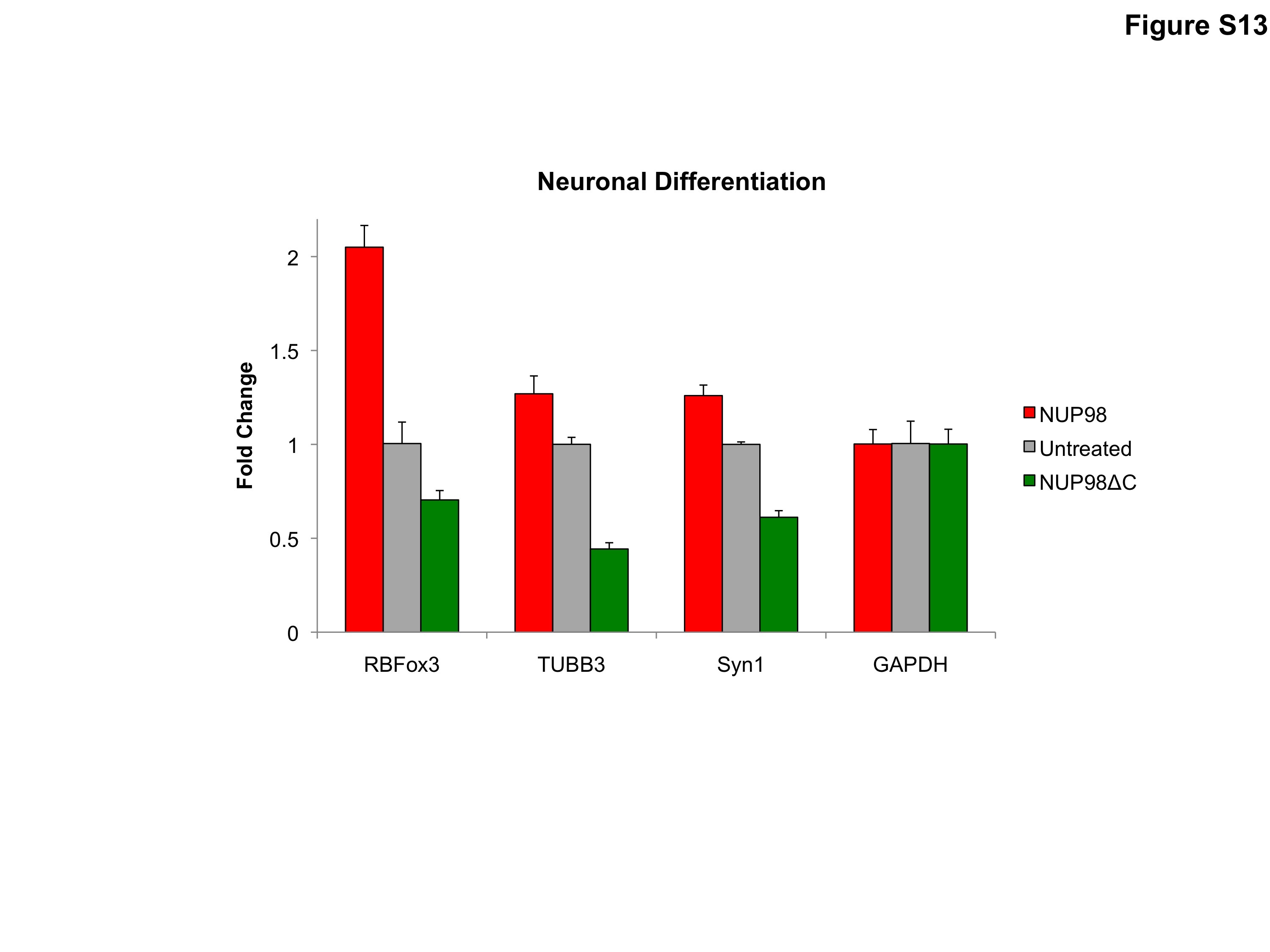

Supplement: Figure S13 — NUP98 regulates neuronal differentiation. Fold change in expression levels of markers for differentiated neurons in cells overexpressing full length NUP98 (NUP98) or its dominant negative fragment (NUP98ΔC), compared to Untreated control. Neural progenitor cells were mock-infected (Untreated), infected with lentiviruses encoding full length NUP98 (NUP98) or the dominant negative fragment of NUP98 (NUP98ΔC), and differentiated into post-mitotic neurons over a month's course. RNAs were extracted from cells at the end of 1 month's differentiation and RT-qPCR was performed for indicated differentiation markers and GAPDH as a negative control. (JPG) [file pgen.1003308.s013.jpg]
