# Supplementary material for: Dynamic Association of NUP98 with the Human Genome
Source: PLoS Genet. 2013 Feb 28;9(2):e1003308. doi: 10.1371/journal.pgen.1003308 (PMC3585015; doi:10.1371/journal.pgen.1003308)
Supplement: Text S1 — Inventory of Supplemental Information and Experimental Procedures. (DOCX) [file pgen.1003308.s014.docx]

**Supplemental Information**

**Inventory of Supplemental Information**

**Supplemental Figure Legends**

Figure S1. Validation of ChIP-Seq method

Figure S2. Number of reads from ChIP-Seq experiments

Figure S3. Differentiation of human embryonic stem cells into neural progenitor cells

Figure S4. Examples of cell type specific NUP98-binding regions

Figure S5. Over-represented transcription factor motifs in NUP98-binding regions

Figure S6. Over-represented transcription disease terms in NUP98-binding regions

Figure S7. NUP98 associates with distinct subsets of active and silent genes in embryonic stem cells

Figure S8. NUP98 or fragment overexpression did not affect expression levels of non-NUP98 binding genes

Figure S9. Localization of overexpressed NUP98 and its fragment in neural progenitor cells

Figure S10. NUP35 overexpression did not affect expression levels of NUP98 binding genes

Figure S11. Gene localization of two groups of NUP98 targets

Figure S12. Analysis of neighboring genes of NUP98 targets regarding NUP binding and intranuclear localization

Figure S13. NUP98 regulates neuronal differentiation

**Supplemental Experimental Procedures**

Cell culture and differentiation

Chromatin immunoprecipitation (ChIP)

Analysis of ChIP-Seq data

Immunofluorescence (IF)

IF-FISH

RNA extraction and qRT-PCR

**Supplemental Figure Legends**

**Figure S1. Validation of ChIP-Seq method**

(A) IMR90 cells were stained with two independent anti-human NUP98 antibodies NUP98 Ab1 and NUP98 Ab2 (green), mAb414 (red), and Hoechst (blue).

(B) IMR90 cells with scrambled RNAi (scrRi) or *NUP98* RNAi (*NUP98*Ri) were lysed according to the ChIP-Seq protocol and proteins in the lysate were transferred to membrane and blotted with two NUP98 antibodies used for ChIP-Seq (NUP98Ab1 and NUP98Ab2). GAPDH was used as loading control.

(C) Immunoprecipitation was performed according to the ChIP-Seq protocol using normal rabbit IgG (IgG) or two NUP98 antibodies (NUP98Ab1, NUP98Ab2) and immunoblotted with NUP98 antibody.

(D) Chromosomal view of NUP98 binding regions on chromosome 1 from four independent ChIP-Seq experiments using two NUP98 antibodies under formaldehyde crosslinking condition(NUP98Ab1-F, NUP98Ab2-F) and under formaldehyde-disuccinimidyl glutarate double crosslinking condition (NUP98Ab1-FD, NUP98Ab2-FD).

(E) Overlap between NUP98 binding regions from ChIP-Seq experiments using two NUP98 antibodies (NUP98Ab1, NUP98Ab2).

(F) Example of peak calling using the Genomatix software. Reads from two NUP98 antibody ChIP-Seq experiments (NUP98Ab1 and NUP98Ab2) and normal rabbit IgG ChIP-Seq experiment (IgG) were shown. Region called as peak by the Genomatix software was indicated by the block in blue (NUP98 Peak).

(G) Randomly selected seven ChIP-Seq peaks (T1 from T7) called by Genomatix and two non-NUP98 binding regions (NC1 and NC2) were tested for NUP98 binding by target ChIP-qPCR using independent batch of IMR90 cells and independent lot of NUP98 antibody. Error bars were computed as standard deviation from triplicates. P value was obtained from Student’s t-test and comparisons with P value <0.05 indicated with asterisks.

**Figure S2. Number of reads from ChIP-Seq experiments**

Number of total reads and mappable reads obtained from each ChIP-Seq experiment.

**Figure S3. Differentiation of human embryonic stem cells into neural progenitor cells**

(A) Scheme showing differentiation of human embryonic stem cells (HESCs) into Embryoid Bodies (EBs), neural rosettes and neural progenitor cells (NeuPCs). The neural progenitor cell cultures are grown as monolayers after neural rosette dissociation.

(B) Markers for homogeneous NPC population (Nestin and Sox2) at lower (upper panel) and higher (lower panel) magnification.

(C) Quantification of percentage of cells expressing a characteristic neuroprogenitor marker, Nestin. Human embryonic stem cells typically do not express Nestin in contrast to differentiated populations of neural progenitor cells that show homogenous expression of Nestin.

**Figure S4. Examples of cell type specific NUP98-binding regions**

Reads from NUP98 ChIP-Seq experiments were shown for embryonic stem cells (ESC), neural progenitor cells (NeuPC), neurons (Neuron), and IMR90 cells (IMR90). Peak assigned were indicated in blue. Transcriptional start sites as from the Genomatix database were shown in red. Peaks found in ESCs, NeuPCs and IMR90 cells were shown in (A), (B), and (C), respectively.

**Figure S5. Over-represented transcription factor motifs enriched in NUP98-binding regions**

(A and B) GA-boxes were over-represented in NUP98-binding genes (A) and NUP98 binding promoters (B) in ESCs and NeuPCs.

(C) Over-represented transcription factor motifs in NUP98-binding regions in ESCs and NeuPCs. Transcription factor motifs were ranked by Z-score and motifs with Z-score more than 10 were listed.

**Figure S6. Over-represented disease terms enriched in NUP98-binding regions**

Disease terms enriched in NUP98 binding genes in NeuPCs by MeSH term analysis.

**Figure S7. NUP98 associates with distinct subsets of active and silent genes in embryonic stem cells**

(A) Pearson’s correlation between pairs of histone modifications for NUP98 binding regions in ESCs. Histone modification levels were calculated from ([Lister et al. 2011](#_ENREF_34)), GSM605321, and GSM605309.

(B, C, and D) For each histone modification type, NUP98 binding genes were ranked by their histone modification levels and top 40% genes were selected for gene ontology analysis. Biological process categories that are uniquely enriched for specific histone modification types were shown in red for active histone marks and in blue for silent histone mark.

(E, F, G, and H) Expression levels of NUP98 binding genes that were high in each of the four histone modifications were compared to those of same number of randomly selected genes. P values were obtained by Mann-Whitney U tests. Top and bottom of the boxes in the plot are 25^th^ and 75^th^ percentile, centerline is the 50^th^, and whiskers extend to 1.5 interquartile range from the upper and lower quantile.

**Figure S8. NUP98 or fragment overexpression did not affect expression levels of non-NUP98 binding genes**

(A) Fold change in expression levels of non-NUP98 binding genes upon NUP98 overexpression in NeuPCs. Error bars were computed as standard deviation from triplicates.

(B) Fold change in expression levels of non-NUP98 binding genes upon NUP98ΔC fragment overexpression in NeuPCs. Error bars were computed as standard deviation from triplicates.

**Figure S9. Localization of overexpressed NUP98 and its fragment in neural progenitor cells**

(A) Live cell images of neural progenitor cells electroporated with plasmids encoding GFP-tagged full length NUP98 (NUP98-GFP), GFP-tagged NUP98 fragment (NUP98ΔC-GFP), and mock transfected (untreated). GFP channel (left) and phase (right) images were shown.

(B) High magnification confocal images of neural progenitor cells fixed after electroporation with plasmids encoding GFP-tagged full length NUP98 (NUP98-GFP) and GFP-tagged NUP98 fragment (NUP98ΔC-GFP). Cells were stained with the nuclear pore marker 414 in red, anti-GFP antibody in green, and Hoechst in blue. In the overlay pictures, a line was drawn across the nuclei and 414 and GFP signals were plotted along the line.

**Figure S10. NUP35 overexpression did not affect expression levels of NUP98 binding genes**

(A) Fold change in expression levels upon NUP35 overexpression in NeuPCs. Error bars were computed as standard deviation from triplicates.

(B) Western blot GAPDH and GFP in NeuPCs with overexpression of GFP-NUP35 or

untreated condition as negative control.

**Figure S11. Gene localization of two groups of NUP98 targets**

Representative 3D IF-FISH images showing the localization of group I genes (*NRG1* and *MAP2*, in A) and group II genes (*SOX5* and *ERBB4*, in B) through development, i.e. in ESC, NeuPC, and Neuron. FISH probes were shown in red, LMNB staining in green, and Hoechst in blue. Each set of images includes the x-y, y-z and x-z planes that cross at the FISH probe signal.

**Figure S12. Analysis of neighboring genes of NUP98 targets regarding NUP binding and intranuclear localization**

(A) In neural progenitor cells, group I (GRIK1, NRG1, and MAP2) and group II (GPM6B, SOX5, ERBB4) NUP98 targets were tested for NUP98 and NUP133 binding by ChIP-qPCR. For each group I genes, two neighboring genes, one within reported lamin-associated domain (from UCSC genome browser) and one outside of lamin-associated domain, were also analyzed for NUP98 and NUP133 binding (USP16, CLDN17, DCTN16, WRN, KCF7 and PTH2R). GAPDH and ACT genes were used as additional negative control. Error bars were computed as standard deviation from triplicates.

(B) Percentage of selected genes localized at the nuclear periphery from IF-FISH experiments in neural progenitor cells.

**Figure S13. NUP98 regulates neuronal differentiation**

Fold change in expression levels of markers for differentiated neurons in cells overexpressing full length NUP98 (NUP98) or its dominant negative fragment (NUP98ΔC), compared to Untreated control. Neural progenitor cells were mock-infected (Untreated), infected with lentiviruses encoding full length NUP98 (NUP98) or the dominant negative fragment of NUP98 (NUP98ΔC), and differentiated into post-mitotic neurons over a month’s course. RNAs were extracted from cells at the end of 1 month’s differentiation and RT-qPCR was performed for indicated differentiation markers and GAPDH as a negative control.

**Supplemental Experimental Procedures**

**Cell Culture and Differentiation**

Human embryonic stem cell line HUES6 was grown in mTeSR1 medium (STEMCELL Technologies). To obtain neuroprogenitor cells, embryoid bodies were formed by mechanically lifting HUES6 cells and transferring them to low-adherence dishes in HUES6 medium without FGF2 for approximately 7 days. After that, embryoid boides were plated onto poly-ornithine/laminin (Sigma)-coated dishes in DMEM/F12 supplemented with N2 and B27 (Invitrogen). Rosettes were visible to collect after 5-7 days. Rosettes were then dissociated with TripLE (Invitrogen) diluted 1:5 in PBS and plated again onto poly-ornithine/laminin-coated dishes with the same medium but now with the addition of 10ng/ml FGF2. Homogeneous populations of neuroprogenitor cells were achieved after 1-2 passages with TripLE in the same conditions. HUES6-derived neural progenitor cells were grown in DMEM/F12/Glutamax (Invitrogen) supplemented with N-2 (Invitrogen), B-27 (Invitrogen), 20ng/ml FGF2 (Stemgent), and 1μg/ml laminin (Invitrogen). For differentiation into neurons, neural progenitor cells were plated at low density and cultured in DMEM/F12/Glutamax supplemented with N-2, B-27, 20ng/ml BDNF (Brain-derived neurotrophic factor, Peprotech), 20ng/ml GDNF (Glial-derived neurotrophic factor, Peprotech) and 1μg/ml laminin for 1 month. Early passage IMR90 cells were grown in DMEM (Mediatech), 15% FBS (Invitrogen) and MEM nonessential amino acids (Mediatech).

**Chromatin immunoprecipitation (ChIP)**

For formaldehyde crosslinking, cells were fixed in 1% formaldehyde (Polysciences) for 10min. For formaldehyde-disuccinimidyl glutarate crosslinking, cells were fixed in 2mM disuccinimidel glutarate (Pierce) for 30min and then fixed in 1% formaldehyde for 10min. Fixation was stopped by adding glycine to a final concentration of 125mM. Fixed cells were lysed by Lysis/Wash Buffer (150mM NaCl, 5mM EDTA, 50mM Tris pH7.5, 0.5% NP-40) and spun down. Pellet was resuspended in Shearing Buffer (1% SDS, 10mM EDTA, 50mM Tris pH8.0) and sonicated to DNA fragments of sizes between 150-300 base pairs using Bioruptor (Diagenode). Sheared chromatin was diluted 10 fold using Dilution Buffer (0.01% SDS, 1.1% TX-100, 1mM EDTA, 16.7mM Tris pH8.0, 167mM NaCl) and incubated with primary antibody overnight at 4°C. Antibody-chromatin mixtures were then incubated with pre-blocked Dynabeads M280 sheep-anti-rabbit IgG for 6 hours at 4°C. Beads were collected, washed with Lysis/Wash Buffer for 6 times and DNA was eluted, de-crosslinked, treated with RNase and protease, and column purified. Purified DNA was subjected to qPCR analysis or ChIP-Seq library construction. NUP98Ab1 was rabbit anti-human NUP98 polyclonal antibody (Cell Signaling 2292), and NUP98Ab2 was rabbit anti-human NUP98 monoclonal antibody (Cell Signaling 2598). NUP98Ab1 and formaldehyde crosslinking was used for ChIP-Seq experiments in ESCs and NeuPCs.

**Analysis of ChIP-Seq data**

**Peak assignment to genes or promoters.** Genomic positions of exons/introns were determined by the mapping of cDNAs from different sources (e.g.RefSeq, GenBank, Ensembl) in Genomatix’s transcript collection to the reference genome, and promoter was defined as 500bp/100bp up/downstream of the transcriptional start site. Peak was assigned to promoters if it overlapped with any genomic regions defined as promoters, and to genes if it overlapped with promoters/exons/introns.

**Correlation with gene expression levels.** Gene expression values as used in Figure 3A,B,C,E were normalized expression/enrichment value (NE-value) obtained using the expression analysis for RNASeq data in the Genomatix software. The NE-value was calculated based on the formula: NE = c * #reads_region_ / (#reads_mapped_ * length_region_), where #reads_region_ was the number of reads (sum of base pairs) of falling into the transcript, #reads_mapped_ was the number of all mapped reads (in base pairs), length_region_ was the transcript length in base pairs, and c was a normalization constant set to 10^7^.

**Correlation with histone modification levels.** For given selected genomic region (NUP98-binding or randomly selected region), histone modification level (as shown in Figure 4 and S6E,F,G,H) was calculated as the number of reads falling into the region from histone modification ChIP-Seq experiment normalized to that from input of ChIP-Seq experiment by subtraction.

**Immunofluorescence (IF)**

Cells were fixed in PBS/4%PFA for 5min, blocked in IF Buffer (PBS, 1%BSA, 0.1%TX-100) for 20min, incubated with primary antibody for 1 hour, washed 3 times in IF Buffer, incubated with secondary antibody for 1 hour, washed in IF Buffer, stained with Hoechst and mounted in Vectashield.

**IF-FISH**

Cells were fixed, stained according to the immunofluorescence procedures, fixed again with 4% PFA for 10min, washed with 2xSSC, permeablized in 0.1M HCl, 0.7% TX-100 on ice for 15min, washed with 2xSSC, denatured in 50% formamide, 2xSSC pH7.2 for 30min at 80°C, and washed with ice-cold 2xSSC. While cells were prepared for hybridization, 200ng of DIG-labeled FISH probes were combined with 5ul human Cot-I DNA (Invitrogen) and 10ug salmon sperm DNA, precipitated, resuspended and incubated with 5ul 100% formamide for 30min at 37°C, denatured for 8min at 85°C, incubated for 1hr at 37°C and combined with 5ul 2xhybridization buffer (4xSSC, 20% dextran sulfate, 2mg/ml BSA). Cells were then incubated with probes overnight at 42°C, re-blocked, stained with anti-DIG antibody (Roche) and Hoechst and mounted in Vectashield. Three-dimensional image stacks were recorded with Zeiss LSM710 scanning scope using a 63x objective, 512x512 resolution, 2x averaging and optimal interval (0.31μm) between stacks in Z-direction and three-dimensional images were reconstructed from the Z-stack images.

**RNA Extraction and qRT-PCR**

RNA was extracted from cells using Trizol (Ambion) and column purified using RNeasy kit (Qiagen). RNA was subsequently reverse transcribed using the QuantiTect reverse transcription kit (Qiagen) and cDNA was subjected to qPCR using SYBR Green PCR Master Mix (AppliedBiosystems). Primer sequences are as follows:

GAPDH-f GTCAGCCGCATCTTCTTTTG

GAPDH-r ACGACCAAATCCGTTGACTC

GRIK1-f ATCAGAGCTGATGCCCAAAG

GRIK1-r ATGGGGGATTCCATTCTCTC

NRG1-f TTTTCCCAAACCCGATCC

NRG1-r CTTTGCCTCTGCCTTCTTTG

MAP2-f CTTTACTTCCCTTCCGCTTC

MAP2-r CTCCGGAGAAAAAGGAGAGG

GPM6B-f GGGGAGTCCAAAAGAAAAGG

GPM6B-r ATGGCTGGCTTCATACCATC

SOX5-f TTGCCAGCCAGCTTAGAATC

SOX5-r AACTGTCCTGTTTGGGATCG

ERBB4-f GGGGATATGCCATTTGGAC

ERBB4-r ACCCAGACCCAAAGTCCTG

ARHGAP26-f TGCAGTGTTCAGCCTCCTCGTCA

ARHGAP26-r CCTTCCGGAACGGTGTGCTGG

ZBTB16-f GAACTGCCGGGCGATGCTGT

ZBTB16-r GGCCAATAGAGGAGGCGCAGC

DCC-f TTCTGACGTGCCAAGTGCCCC

DCC-r TCTCACCCCTGCGGGTCGTC

ROBO1-f TCGCCCAGGGCAGCCTACTC

ROBO1-r TTCTCGCCAGAGTCCCCGCT

SEMA6D-f GGAAGAGTCAATTTTGCTGAGCCCC

SEMA6D-r TGCCACTGAGCTACCTTCCTCCT

NRXN1-f GAGGGAGCGGGAGCCCTTCA

NRXN1-r GCTGTTGGGCGGCTCATCGT

POU6F2-f AATCCGGCGCCTGTCCCTTG

POU6F2-r GGTGTGTCTGCAGATGGCCGA

IGF1R-f GCCTGACAACGACACGGCCT

IGF1R-r ACAGCGCCAGCCCTCAAACC

TBX19-f TGTGACCAAGAATGGCAGACGGA

TBX19-r CTTGCCAGCGGGCACCCATT

SEMA3A-f TTCAACGGCTGGGGTTGCCC

SEMA3A-r TGTGCGTCTCTTTGCAGTGGGAA

FGF12-f CCCACTTCGCGCCTCTCGGA

FGF12-r CGGCCCGCGGTTCTGAAGATT

FGF1-f AGGTACAGCCCTCGGCCTACA

FGF1-r TCGGTCAGGGCTGTGAAGGTGG

PARD3-f ACCAGCTGTCCCCTACAGTGAAT

PARD3-r GCATCTGCCGTCACAAACCCCA

NRP2-f TAAAGGGGGCACCCTCCTGCC

NRP2-r GCGGAACCGGTGGTAGTGGA

ZNF423-f CGGGCCTGCCATACCCTTGC

ZNF423-r CGGCAGCTTGTCGCTGTGGA

GRIP1-f GGGGCTGCCGATAGTGCAGAG

GRIP1-r CACACGCCTGTCAGCTTTCTCCT

DSCAM-f TCGACACCAGCGGGGACAGT

DSCAM-r GAGAAGAAGGCCCCGTGCCG

FYN-f TGCGCCATCTGTCAGGAGCG

FYN-r CTCGCCTCTACTCTCGCCGC
